# Perioperative sintilimab and neoadjuvant anlotinib plus chemotherapy for resectable non-small-cell lung cancer: a multicentre, open-label, single-arm, phase 2 trial (TD-NeoFOUR trial)

**DOI:** 10.1038/s41392-024-01992-0

**Published:** 2024-10-28

**Authors:** Hongtao Duan, Changjian Shao, Zhilin Luo, Tianhu Wang, Liping Tong, Honggang Liu, Xin Yao, Jie Lei, Jinbo Zhao, Yuan Gao, Tao Jiang, Xiaolong Yan

**Affiliations:** 1 Department of Thoracic Surgery, Tangdu Hospital, Air Force Medical University, No. 1, Xinsi Road, Xi’an, Shaanxi China; 2 Department of Thoracic Surgery, Third Affiliated Hospital, Chongqing Medical University, Chongqing, China; 3 State Key Laboratory of Holistic Integrative Management of Gastrointestinal Cancers, Biotechnology Center, School of Pharmacy, Air Force Medical University, No. 169, Changle West Road, Xi’an, Shaanxi China

**Keywords:** Drug development, Lung cancer, Tumour biomarkers

## Abstract

This open-label, single-arm, phase 2 trial evaluated the efficacy and safety of neoadjuvant sintilimab combined with anlotinib and chemotherapy, followed by adjuvant sintilimab, for resectable NSCLC. Forty-five patients received anlotinib (10 mg, QD, PO, days 1–14), sintilimab (200 mg, day 1), and platinum-based chemotherapy of each three-week cycle for 3 cycles, followed by surgery within 4–6 weeks. Adjuvant sintilimab (200 mg) was administered every 3 weeks. The primary endpoint was achieving a pathological complete response (pCR). From June 10, 2021 through October 10, 2023, 45 patients were enrolled and composed the intention-to-treat population. Twenty-six patients (57.8%) achieved pCR, and 30 (66.7%) achieved major pathological response (MPR). Forty-one patients underwent surgery. In the per-protocol set (PP set), 63.4% (26/41) achieved pCR, and 73.2% achieved MPR. The median event-free survival was not attained (95% CI, 25.1-NE). During the neoadjuvant treatment phase, grade 3 or 4 treatment-related adverse events were observed in 25 patients (55.6%), while immune-related adverse events were reported in 7 patients (15.6%). We assessed vascular normalization and infiltration of immune-related cells by detecting the expression of relevant cell markers in NSCLC tissues with mIHC. Significant tumor microenvironment changes were observed in pCR patients, including reduced VEGF^+^ cells and CD4^+^Foxp3^+^ Treg cells, and increased perivascular CD4^+^ T cells, CD39^+^CD8^+^ T cells, and M1 macrophages. In conclusion, perioperative sintilimab and neoadjuvant anlotinib plus chemotherapy achieved pCR in a notable proportion of patients with resectable NSCLC and were associated with profound changes in the tumour microenvironment (ClinicalTrials.gov NCT05400070).

## Introduction

Lung cancer ranks as the second most common cancer and is the leading cause of cancer-related deaths worldwide.^1^ In China alone, it accounts for 40% of global lung cancer fatalities.^2^ Approximately 20–25% of patients with non-small cell lung cancer (NSCLC) present with resectable disease at diagnosis.^3^ Compared to surgery, standard neoadjuvant chemotherapy offers only limited improvements in recurrence-free survival (RFS) and overall survival (OS).^4^ Key studies, such as the Checkmate-816^5^ and KEYNOTE-671^6^ trials, have demonstrated that combining neoadjuvant immunotherapy with concurrent chemotherapy is both effective and safe for resectable NSCLC. Though perioperative immune checkpoint inhibition holds promise for improving response rates and decreasing recurrence in patients with resectable NSCLC,^7^ the majority of patients with resectable disease fail to achieve a pathological complete response (pCR). In the randomized TD-FOREKNOW trial, patients with resectable stage IIIA or IIIB NSCLC who were treated with camrelizumab and chemotherapy had a pCR rate of 32.6%, compared to an 8.9% pCR rate in those receiving chemotherapy alone.^8^ In the Checkmate-816 trial, ~24.0% of patients with resectable stage IB–IIIA NSCLC who received neoadjuvant nivolumab in combination with chemotherapy achieved a pCR,^5^ while in the KEYNOTE-671 trial, 18.1% of resectable stage II–IIIB NSCLC patients achieved a pCR to perioperative pembrolizumab.^6^

Aberrant oncoangiogenesis is a hallmark of cancer and a key driver of tumour growth and metastasis^9^ and represents a rational target in NSCLC,^10^ as demonstrated in the IMpower150 study^11^ and the TASUKI-52 trial.^12^ Antiangiogenic therapy can shift the immunosuppressive tumour microenvironment (TME) towards an immune-active state. Nevertheless, neoadjuvant bevacizumab plus chemotherapy failed to improve the rate of pathological downstaging over historical control in patients with resectable stage IB–IIIA nonsquamous NSCLC.^13^ The findings from trials of neoadjuvant immunotherapy or antiangiogenic therapy plus chemotherapy indicate that either immunotherapy or antiangiogenic therapy alone leaves many patients at risk for relapse and eventual death, while a therapeutic strategy combining the two approaches can lead to a better clinical outcome.^14^

Sintilimab is a selective, fully human IgG4 anti-PD-1 monoclonal antibody, approved in China for the treatment of locally advanced or metastatic NSCLC. In the neoadjuvant setting, sintilimab monotherapy led to a major pathological response (MPR) in 15 resectable stage IA–IIIB NSCLC patients (15/37, 41%) and pCR in 6 (6/37, 16%). When added to chemotherapy, neoadjuvant sintilimab attained a MPR rate of 43% (13/30) in resectable stage IIIA NSCLC patients.^15^ In an interim analysis of a phase 2 trial, neoadjuvant sintilimab plus chemotherapy yielded an MPR of 63% (10/16) and a pCR of 31% (5/16) in patients with resectable stage IIIA/B NSCLC.^16^

Anlotinib is an antiangiogenic drug and multitargeted receptor tyrosine kinase inhibitor that targets VEGFR1, VEGFR2, VEGFR3, c-KIT, and PDGFR β.^17^ It is approved in China for treating locally advanced and metastatic NSCLC that has progressed or recurred after at least two lines of systemic chemotherapy.^18^ Compared with intravenous infusion, multikinase inhibitors provide more convenient oral dosing for currently available antiangiogenic agents. Treatment with anlotinib plus the programmed death ligand 1 (PD-L1) immune checkpoint inhibitor TQB2450 increased the progression-free survival (PFS) of pretreatment stage IIIB or IV NSCLC patients with no mutated driver genes by 6 months (total: 8.7 months, 95% confidence interval [CI] 6.1–17.1) vs. TQB2450 2.8 months, 95% CI 1.4–4.7). In a phase 1b study, 16 treatment-naïve patients (16/22, 73%) with unresectable, driver-negative, stage IIIB/C or IV NSCLC showed an objective response to anlotinib plus sintilimab.^19^ Anlotinib has not yet been studied alone or in combination with other treatments, including immune checkpoint inhibitors, for resectable NSCLC in clinical trials.

This phase 2 trial evaluated the efficacy and safety of neoadjuvant sintilimab combined with anlotinib and chemotherapy, followed by adjuvant sintilimab, for the treatment of resectable NSCLC. Additionally, it explored alterations in the TME as potential biomarkers for predicting the response to this combined neoadjuvant immunotherapy and antiangiogenic treatment.

## Results

### Patient baseline and treatment characteristics

From June 10, 2021 through October 10, 2023, 67 patients underwent screening, 45 of whom, including 41 males and 4 females, were eligible to receive sintilimab plus anlotinib concurrent with platinum-based doublet chemotherapy; these patients composed the intention-to-treat (ITT) population (Supplementary Fig. 1). Among them, 34 (75.6%) had squamous cell carcinoma and 33 (73.3%) had stage III disease (IIIA, *n* = 15, 33.3%, and IIIB, *n* = 18, 40.0%). Thirty-seven patients (82.2%) were former or current smokers. The demographics and baseline characteristics of the patients are detailed in Table 1.

**Table 1:** Demographics and baseline characteristics of the intention-to-treat population (*N* = 45)

Variable	Value
Age, years	
Median (range)	60 (52–74)
≥60	28 (62.2)
Male sex	41 (91.1)
Tumour stage	
T1	4 (8.8)
T2	8 (17.8)
T3	16 (35.6)
T4	17 (37.8)
Nodal stage	
N0	11 (24.4)
N1	10 (22.2)
N2	24 (53.3)
Clinical stage	
IIA	4 (8.8)
IIB	8 (17.8)
IIIA	15 (33.3)
IIIB	18 (40.0)
Histological status	
Squamous cell carcinoma	34 (75.6)
Adenocarcinoma	10 (22.2)
Sarcomatoid carcinoma	1 (2.2)

Data are expressed as number (%) unless otherwise specified

In the neoadjuvant treatment period, one patient discontinued treatment due to grade 3 elevated aminotransferases and grade 4 myelosuppression. Three patients declined surgery. In the PP set, 33 of 41 patients (80.5%) completed the prespecified 3 cycles of neoadjuvant treatment, and 8 patients underwent surgery after 2 cycles of treatment. Six patients discontinued treatment due to adverse events (AEs) in the adjuvant treatment period. The patients’ exposure to anlotinib, sintilimab and individual chemotherapeutic drugs is summarized in the Supplementary Materials (specific circumstances of the patients).

### Efficacy measures

**Fig. 1:**
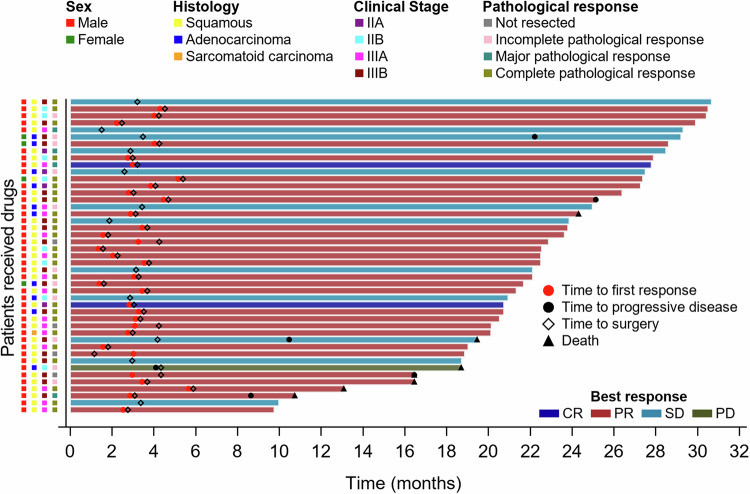
Responses to sintilimab combined with anlotinib and concurrent neoadjuvant chemotherapy were assessed in patients with resectable non-small-cell lung cancer within the intention-to-treat population. Investigators evaluated patients based on the Response Evaluation Criteria in Solid Tumours (RECIST), version 1.1. Each swim lane corresponds to a single patient in the ITT group, with patient characteristics and outcomes indicated by specific color codes. CR complete response, PR partial response, SD stable disease, PD progressive disease

In the ITT population, pCR and MPR were reported for 26 patients (57.8%, 95% CI 43.3–71.0) and 30 patients (66.7%, 95% CI 52.1–78.6), respectively. Two patients achieved CR, and 30 achieved a partial response (PR). The objective response rate (ORR) was 71.1% (32/45, 95% CI 55.7–83.6) (Fig. 1). Twelve patients had stable disease (SD); the disease control rate (DCR) was 97.8% (44/45, 95% CI 88.2–99.9) (Supplementary Fig. 2 and Table 2). Two patients with squamous cell carcinoma achieved CR, and 24 achieved PR (Supplementary Fig. 3a). Their ORR was 76.5% (26/34, 95% CI 60.0–87.6). Eight of these patients had SD; the DCR was 100.0% (34/34, 95% CI 89.6–100.0). No patients with adenocarcinoma achieved CR, while 5 achieved PR. Their ORR was 50.0% (5/10, 95% CI 23.7–76.3) (Supplementary Fig. 3b). Four of them had SD, and the DCR was 90.0% (9/10, 95% CI 59.6–98.2).

**Table 2:** Treatment responses of the study patients

	Intension-to-treat population				Per-protocol set			
	All, *N* = 45	Squamous cell carcinoma, *N* = 34	Adenocarcinoma, *N* = 10	Sarcomatoid carcinoma, *N* = 1	All, *N* = 41	Squamous cell carcinoma, *N* = 30	Adenocarcinoma, *N* = 10	Sarcomatoid carcinoma, *N* = 1
Pathologic responses, *n/N* (%, 95% CI)								
Pathological complete response	26/45 (57.8, 43.3–71.0)	22/34 (64.7, 47.9–78.5)	3/10 (30.0, 10.8–60.3)	1/1 (100.0, 20.6–100.0)	26/41 (63.4, 48.1–76.4)	22/30 (73.3, 55.6–85.8)	3/10 (30.0, 10.8–60.3)	1/1 (100.0, 20.6–100.0)
Major pathological response	30/45 (66.7, 52.1–78.6)	26/34 (76.5, 60.0–87.6)	3/10 (30.0, 10.8–60.3)	1/1 (100.0, 20.6–100.0)	30/41 (73.2, 58.1–84.3)	26/30 (86.7, 70.3–94.7)	3/10 (30.0, 10.8–60.3)	1/1 (100.0, 20.6–100.0)
Clinical responses, *n/N* (%, 95% CI)								
CR	2	2	0	0	2	2	0	0
PR	30	24	5	1	30	24	5	1
ORR	32/45 (71.1, 55.7–83.6)	26/34 (76.5, 60.0–87.6)	5/10 (50.0, 23.7–76.3)	1/1 (100.0, 20.6–100.0)	32/41 (78.0, 63.3–88.0)	26/30 (86.7, 70.3–94.7)	5/10 (50.0, 23.7–76.3)	1/1 (100.0, 20.6–100.0)
SD	12	8	4	0	8	4	4	0
DCR	44/45 (97.8, 88.2–99.9)	34/34 (100.0, 89.6–100.0)	9/10 (90.0, 59.6–98.2)	1/1 (100.0, 20.6–100.0)	40/41 (97.6, 87.4–99.6)	30/30 (100, 88.7–100.0)	9/10 (90.0, 59.6–98.2)	1/1 (100.0, 20.6–100.0)
PD	1	0	1	0	1	0	1	0

Data are expressed as number (%) unless otherwise specified

Pathological complete response is defined as the absence of residual tumour cells in resected primary tumour and lymph nodes [ypT0N0M0]) and major pathological response is defined as ≤10% viable tumour cells in resected primary tumour and lymph nodes as assessed by a pathologist. Clinical responses were assessed by the investigator according to the Response Evaluation Criteria in Solid Tumors (RECIST), version 1.1

In the PP set, pCR occurred in 26 patients (26/41, 63.4%, 95% CI 48.1–76.4), and MPR occurred in 30 patients (30/41, 73.2%, 95% CI 58.1–84.3) (Fig. 1 and Table 2). Pathologically downstaged tumours were detected in 87.8% (36/41) of the patients. pCR occurred in 73.3% (22/30, 95% CI 55.6–85.8) of the patients with squamous cell carcinoma (Supplementary Fig. 3c) and 30.0% (3/10, 95% CI 10.8–60.3) of the patients with adenocarcinoma (Supplementary Fig. 3d). One patient with sarcomatoid carcinoma achieved both a PR and a pCR.

Lobectomy was the most common surgical procedure (Supplementary Table 1). Among patients who underwent surgery, 92.7% (38/41) had complete (R0) resection; the others (3/41, 7.3%) had R1 resection (the lymph node of the highest station was metastatic). None of the patients underwent R2 resection or had unresectable tumours. The median postoperative hospital stay was 8 days, with a range of 3–25 days.

**Fig. 2:**
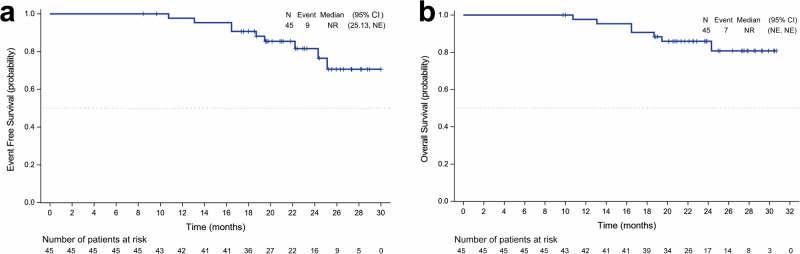
Kaplan–Meier estimates of (**a**) event-free survival and **b** overall survival. EFS was defined as the interval from enrolment to the earliest occurrence of local progression resulting in inoperability; unresectable tumour, disease progression or recurrence according to RECIST version 1.1 as assessed by the investigator; or death from any cause. OS was defined as the time from enrollment to death from any cause. Tick marks represent censored data

As of the data cutoff on December 31, 2023, the median follow-up duration was 22.8 months (IQR 9.9–33.6 months), with a 24-month estimated event-free survival (EFS) rate of 81.5% (95% CI, 64.5–90.9%). The median EFS was not reached (95% CI, 25.1-NE) (Fig. 2a and Supplementary Table 2). A post hoc subgroup analysis revealed no significant differences in EFS among various patient subgroups (Supplementary Table 3). Two patients (4%) were lost to follow-up, and two patients (4%) passed away. The estimated 12-month survival rate was 97.7% (95% CI, 84.6–99.7%). The median OS was not reached (95% CI, NE-NE) (Fig. 2b).

### Safety

All-grade treatment-related AEs (TRAEs), including grade 3 or 4 TRAEs in 25 patients (25/45, 55.6%), occurred in 100.0% (45/45) of patients during the neoadjuvant treatment period. The three most frequent TRAEs (grade 3 or 4) were white blood cell count decrease (5/45, 11.1%), neutrophil count decrease (5/45, 11.1%), and vomiting (4/45, 8.9%) (Table 3). In addition, immune-related AEs (irAEs) occurred in 7 patients (7/45, 15.6%). Two patients developed cavitation-like lesions after anlotinib treatment but did not experience haemoptysis.

**Table 3:** Treatment-related adverse events in the neoadjuvant treatment period (the safety population) and in the adjuvant treatment period (the per-protocol population) and postoperative complications

Variable	Any grade	Grade 3 or 4
Neoadjuvant treatment period		
Any AEs	45 (100.0)	25 (55.6)
White blood cell decreased	35 (77.8)	5 (11.1)
Neutrophil count decreased	34 (75.6)	5 (11.1)
Vomiting	33 (73.3)	4 (8.9)
Alopecia	30 (66.7)	3 (6.7)
Fatigue	27 (60.0)	2 (4.4)
Anaemia	22 (48.9)	1 (2.2)
Nausea	19 (42.2)	1 (2.2)
Leg pain	16 (35.6)	2 (4.4)
Peripheral sensory neuropathy	15 (33.3)	3 (6.7)
Diarrhoea	10 (22.2)	1 (2.2)
Bronchopulmonary haemorrhage	2 (4.4)	–
Rash	5 (11.1)	1 (2.2)
Hypothyroidism	2 (4.4)	2 (4.4)
Pneumonitis	2 (4.4)	–
Alanine aminotransferase increased	1 (2.2)	1 (2.2)
Aspartate aminotransferase increased	1 (2.2)	1 (2.2)
Blood bilirubin increased	1 (2.2)	1 (2.2)
Oral mucositis oral	1 (2.2)	1 (2.2)
Hyperthyroidism	1 (2.2)	–
Adjuvant treatment period		
irAEs	14 (34.1)	7 (17.1)
Adrenal Insufficiency	4 (9.8)	2 (4.9)
Fatigue	4 (9.8)	2 (4.9)
Hypothyroidism	4 (9.8)	1 (2.4)
Rash	2 (4.9)	1 (2.4)
Fever	2 (4.9)	0
Pneumonitis	1 (2.4)	1 (2.4)
Postoperative complications		
Any complications	14 (34.1)	8 (19.5)
Pleural effusion	6 (14.6)	6 (14.6)
Pneumonia	5 (12.2)	3 (7.3)
Heart failure	5 (12.2)	0
Pneumothorax	3 (7.3)	3 (7.3)
Intraoperative blood transfusion	1 (2.4)	0
Hoarseness	1 (2.4)	0
Respiratory failure	0	0
Death within 30 and 90 days	0	0

Data are expressed as number (%)

AEs were assessed using CTCAE. Postoperative complications were evaluated using the Clavien‒Dindo Classification of Surgical Complications

The per-protocol population included all the patients who received at least one dose of sintilimab during the adjuvant treatment period

Treatment-related adverse events were adverse events considered by the investigator to be related to chemotherapy, sintilimab, or anlotinib

In the adjuvant treatment period, irAEs occurred in 14 patients (14/41, 34.1%), including grade 3 irAEs in 7 patients (7/41, 17.1%). Grade 3 adrenal insufficiency and fatigue occurred in 2 patients, respectively (2/41, 4.9%) (Table 3).

According to the Clavien–Dindo classification, 14 patients (14/41, 34.1%) developed postoperative complications. Grade 3 complications, including pleural effusion (6/41, 14.6%), pneumonia (3/41, 7.3%), and pneumothorax (3/41, 7.3%), occurred in 19.5% (8/41) of the patients (Table 3).

### Neoadjuvant anlotinib and sintilimab modulate the TME

Dynamic changes in the tumor microenvironment before and after treatment are vital indicators for evaluating the effectiveness of tumor immunotherapy. Therefore, we focused on evaluating vascular normalization and the infiltration of immune cells relevant to immunotherapy using mIHC technology. Specifically, we evaluated vascular normalization by analyzing the count of VEGF^+^ cells, the ratio of CD31^+^/NG2^+^ cells, and the infiltration of perivascular CD8^+^ and CD4^+^ T cells. Furthermore, we assessed immune cell infiltration by quantifying several immune cell types involved in the tumor immune response, including Treg cells (CD4^+^Foxp3^+^ T cells), M1 macrophages (CD80^+^CD11c^+^ cells), M2 macrophages (CD80^+^CD206^+^ cells), PD-1^+^CD8^+^ cells, and CD39^+^CD8^+^ cells.

Since the existence of Tregs in the TME is closely related to the efficacy of immunotherapy,^20^ we first observed Treg infiltration. CD4^+^Foxp3^+^ Treg cell^21,22^ infiltration decreased significantly after treatment compared to baseline in the pCR group, and there were no significant differences in the non-pCR group (Supplementary Fig. 4a, b). CD8^+^ T cells in the TME are a key cell population in the response to immunotherapy,^23^ so we further examined this population, and the results showed that CD8^+^ T-cell infiltration exhibited an overall upward trend in the pCR group from baseline to posttreatment and a downward trend in the non-pCR group, but the difference was not significant (Supplementary Fig. 4c).

**Fig. 3:**
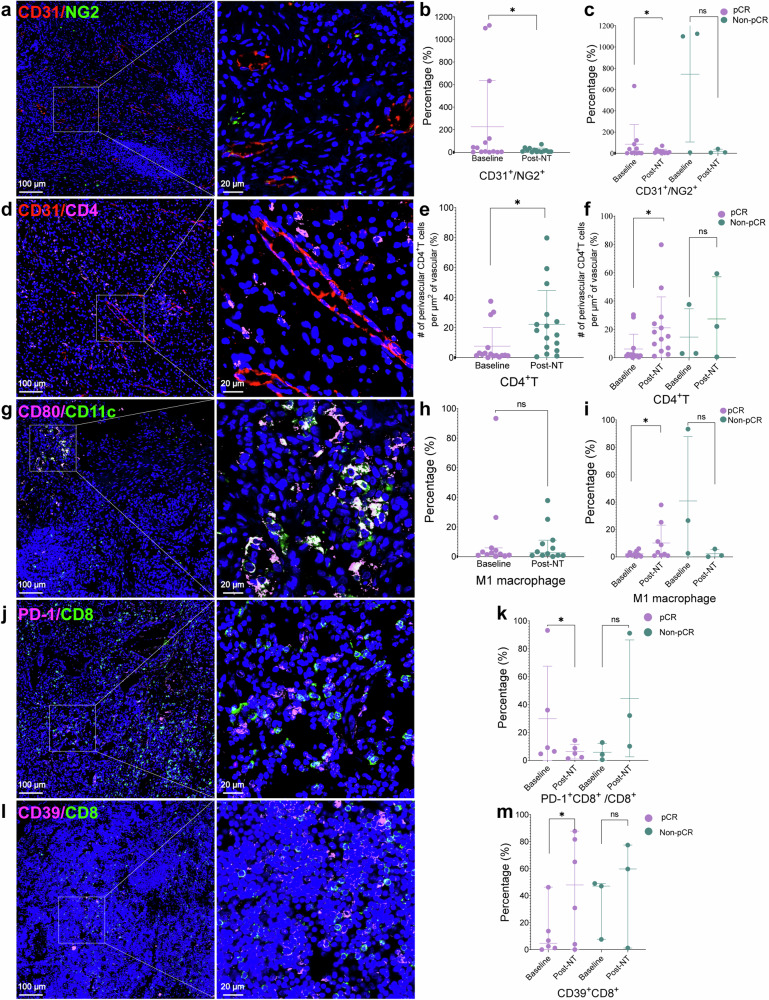
Representative images and quantification of multiplex immunohistochemical staining of tumours at baseline and after neoadjuvant treatment (post-NT). **a**–**c** Assessment of the expression levels of CD31 (endothelial cells, red) and NG2 (pericytes, green) in the lung cancer microenvironment before and after treatment, along with statistical analysis of the ratio of CD31^+^ to NG2^+^. **a** Representative images of the lung cancer microenvironment showing CD31 and NG2 staining. **b** Statistical analysis of the CD31^+^ to NG2^+^ ratio in baseline and post-neoadjuvant treatment samples (paired samples, *n* = 14). **c** Statistical analysis of the CD31^+^ to NG2^+^ ratio in baseline and post-neoadjuvant treatment samples within the pCR group (paired samples, *n* = 11) and the non-pCR group (paired samples, *n* = 3). **d**–**f** Perivascular CD4^+^ T cell count per μm² of vascular area. CD31 staining (red) represents the vascular area, while CD4 staining (pink) indicates the infiltration of CD4^+^ T cells. **d** Representative images of the lung cancer microenvironment showing CD31 and CD4 staining. **e** Statistical analysis of perivascular CD4^+^ T cell count per μm^2^ of vascular area in baseline and post-neoadjuvant treatment samples (paired samples, *n* = 16). **f** Statistical analysis of perivascular CD4^+^ T cell count per μm^2^ of vascular area in baseline and post-neoadjuvant treatment samples within the pCR group (paired samples, *n* = 13) and the non-pCR group (paired samples, *n* = 3). **g**–**i** Assessment of the expression levels of CD80 (pink) and CD11c (green) in the lung cancer microenvironment before and after treatment, along with statistical analysis of the infiltration of CD11c^+^CD80^+^ M1 macrophage. **g** Representative images of the lung cancer microenvironment showing CD80 and CD11c staining. **h** Statistical analysis of the CD11c^+^CD80^+^ M1 macrophage in baseline and post-neoadjuvant treatment samples (paired samples, *n* = 12). **i** Statistical analysis of the CD11c^+^CD80^+^ M1 macrophage in baseline and post-neoadjuvant treatment samples within the pCR group (paired samples, *n* = 9) and the non-pCR group (paired samples, *n* = 3). **j** Representative images of the lung cancer microenvironment showing PD-1 (pink) and CD8 (green) staining. **k** Statistical analysis of the PD-1^+^CD8^+^ T cell in baseline and post-neoadjuvant treatment samples within the pCR group (paired samples, *n* = 5) and the non-pCR group (paired samples, *n* = 3). **l** Representative images of the lung cancer microenvironment showing CD39 (pink) and CD8 (green) staining. **m** Statistical analysis of the CD39^+^CD8^+^ T cell in baseline and post-neoadjuvant treatment samples within the pCR group (paired samples, *n* = 6) and the non-pCR group (paired samples, *n* = 3). Data are mean ± SD. **a**–**h** Wilcoxon paired *t* test. No Significance (ns), ns: *P* > 0.05, *P < 0.05, and **P < 0.01

VEGF plays a crucial role in tumor angiogenesis and contributes significantly to immunosuppression within the TME.^24^ In our observations, patients who achieved a pCR after neoadjuvant therapy showed a notable decrease in VEGF^+^ cells in the TME. Conversely, those who did not achieve pCR exhibited a slight increase in the frequency of VEGF^+^ cells (Supplementary Fig. 5a, b). The ratio of cells that are positive for endothelial cell marker CD31 to cells that are positive for pericyte marker neural/glial antigen (NG2) is a key indicator of vascular normalization.^25^ We found that there was a significant decrease in the CD31^+^/NG2^+^ cell ratio in tumour vessels after neoadjuvant treatment compared with baseline (Fig. 3a, b). Patients who achieved pCR experienced a significant decrease in the frequency of CD31^+^/NG2^+^ cell ratio from baseline, while patients who failed to achieve pCR experienced no significant change from baseline (Fig. 3c).

Since vascular normalization can promote the recruitment of cell populations relevant to the response to immunotherapy,^24,26^ we examined vascular peripheral immune cell infiltration in the TME. We observed a significant increase in perivascular CD4^+^ T cell infiltration post-treatment compared to baseline (Fig. 3d, e). Notably, this increase was significant in the pCR group but not in the non-pCR group (Fig. 3f). There were no significant changes in perivascular CD8^+^ T cell levels after treatment versus baseline in either group (Supplementary Fig. 6a, b).

We also observed a slight upward trend in the overall frequency of M1 macrophages after neoadjuvant treatment (median: 1.98%) versus baseline (median: 1.74%), although the difference was not statistically significant (Fig. 3g, h). Further analysis showed that in the pCR group, the percentage of M1 macrophages increased significantly after neoadjuvant treatment (median: 3.27%) versus baseline (median: 1.36%) (Fig. 3i). In addition, the frequency of M2 macrophages did not significantly change after neoadjuvant treatment compared with baseline in patients in either the pCR or non-pCR group (Supplementary Fig. 7a, b).

We measured the frequency of PD-1^+^ CD8^+^ T cells within CD8^+^ T cells in the TME, as this ratio can predict immunotherapy effectiveness.^27^ In the pCR group, the PD-1^+^ CD8^+^ T cell/CD8^+^ T cell ratio significantly decreased after treatment (4.97%) compared to baseline (9.15%). In contrast, the ratio increased in the non-pCR group after treatment (32.12%) from baseline (4.22%) (Fig. 3j, k).

Immune checkpoint blockade increases the frequency of intratumoral CD39^+^ CD8^+^ T cells, and CD39 may serve as a surrogate marker of tumour-reactive CD8^+^ T cells in human lung cancer.^28^ We found that the frequency of CD39^+^ CD8^+^ T cells was significantly greater after neoadjuvant therapy than at baseline in patients who achieved pCR, while no significant differences were observed in the non-pCR group (Fig. 3l, m).

By analysing the baseline cell subtypes, including the frequencies of CD4^+^Foxp3^+^ Treg cells (Supplementary Fig. 8a), VEGF^+^ cells (Supplementary Fig. 8b), CD39^+^CD8^+^ T cells (Supplementary Fig. 8c), the ratios of PD-1^+^CD8^+^ T cells/CD8^+^ T cells (Supplementary Fig. 8d), M1 macrophages (Supplementary Fig. 8e) and M2 macrophages (Supplementary Fig. 8f), we found that there were no correlations between patient outcomes during neoadjuvant therapy and these cell subtypes. These findings may need validation in larger studies with extended follow-up to statistically correlate survival outcomes with baseline biomarkers.

## Discussion

To the best of our knowledge, this is the first report of perioperative immunotherapy and neoadjuvant antiangiogenic therapy plus SOC chemotherapy for resectable NSCLC. In this phase 2 trial, perioperative immune therapy with sintilimab and neoadjuvant antiangiogenic therapy with anlotinib plus SOC achieved a pCR rate of 57.8% (95% CI 43.3–71.0) in the ITT population and 63.4% in the PP set and a 12-month EFS rate of 97.7% (95% CI 84.6–99.7) in patients with resectable stage IIA-IIIB NSCLC. Furthermore, 73.2% (95% CI 58.1–84.3) of the patients achieved MPR, and the tumours were successfully downstaged in 87.8% of the patients in the PP set. In addition, no safety signals emerged during the trial. There was also no notable increase in the incidence of surgical complications. The results are highly promising, as they demonstrate the feasibility, efficacy, and safety of this combinatorial approach and supporting further exploration of therapeutic regimens for resectable NSCLC.

Although they have not been directly compared with immune therapy plus SOC chemotherapy in the neoadjuvant setting, the proportion of resectable stage IIA-IIIB NSCLC patients who achieved pCR with anlotinib and sintilimab plus SOC chemotherapy (63.4%, 95% CI 48.1–76.4) was higher than that with neoadjuvant nivolumab plus chemotherapy for resectable stage IB–IIIA NSCLC patients in the Checkmate-816 trial (24.0%, 95% CI, 18.0–31.0),^5^ perioperative pembrolizumab for resectable stage II–IIIB NSCLC patients in the KEYNOTE-671 trial (18.1%, 95% CI, 14.5–22.3),^6^ and neoadjuvant sintilimab plus chemotherapy in resectable stage IIIA/B NSCLC patients (31%, 95% CI, 20.4–45.8).^16^ Our results also compare favourably with those of the AEGEAN trial and the Neotorch study. In the AEGEAN trial, the addition of perioperative durvalumab therapy to neoadjuvant chemotherapy and surgery led to a pCR rate of 17.2% (4.3% for placebo; difference, 13.0%; 95% CI, 8.7–17.6) among patients with resectable stage II or III NSCLC.^29^ In the Neotorch study, which was conducted in China, perioperative toripalimab led to significantly more pCR compared with placebo (24.8%, 95% CI, 19.0–31.3 *vs*. 1.0%, 95% CI, 0.1–3.5) in patients with stage III NSCLC.^30^ In addition, the proportion of resectable stage IIA-IIIB NSCLC patients who achieved pCR with anlotinib and sintilimab plus SOC chemotherapy (63.4%, 95% CI 48.1–76.4) was higher than that of patients who achieved pCR with neoadjuvant camrelizumab, an anti-PD-1 antibody and apatinib, an antiangiogenic agent, for resectable stage IIA to IIIB NSCLC patients (15/65, 23%, 95% CI 14–35).^31^ Furthermore, the rate of pathological downstaging reached 87.8% (36/41, 95% CI 74.5–94.7) in our patients. This finding compares favourably to that of neoadjuvant bevacizumab plus chemotherapy for patients with resectable stage IB–IIIA nonsquamous NSCLC^13^ (38%, 95% CI 25–53). Notably, all of the above comparisons were indirect, so clinical trials conducting direct comparisons of different regimens would be more reliable.

pCR and MPR have been associated with better survival outcomes among patients with resectable NSCLC receiving neoadjuvant chemotherapy^32,33^ and among patients receiving neoadjuvant nivolumab plus chemotherapy. The median EFS was not reached, and the 12-month OS was 97.7% (95% CI 84.6–99.7). The high rate of pCR and MPR and the low number of events at 12 months under perioperative sintilimab and neoadjuvant anlotinib for resectable lung tumours are encouraging. In the AEGEAN trial, perioperative durvalumab therapy achieved an EFS of 73.4% (95% CI, 67.9–78.1) at 12 months (64.5% for placebo, 95% CI, 58.8–69.6) among patients with resectable stage II or III NSCLC.^29^ In the Neotorch study, perioperative toripalimab led to significant improvement in EFS (HR = 0.40, 95% CI 0.277–0.565) in patients with stage III NSCLC compared with placebo.^30^ As the data of the current trial are still not mature, it remains to be seen whether improvements in the rate of pCR and the MPR can be translated into survival benefits for resectable NSCLC patients. The results suggest an overall benefit of neoadjuvant antiangiogenic therapy combined with perioperative immunotherapy plus chemotherapy, and the perioperative regimen appears to confer a benefit that is at least similar to, if not greater than, that of neoadjuvant or perioperative immunotherapy alone. Nevertheless, future trials directly comparing neoadjuvant antiangiogenic therapy plus perioperative immunotherapy and perioperative immunotherapy alone are needed.

Compared to chemoimmunotherapy regimens (such as those tested in the KEYNOTE-671 and Checkmate-816 trials), one with an additional antiangiogenic drug, anlotinib, was tested in our study. This antiangiogenic agent has been reported to have a role in promoting vascular normalization.^34,35^ In our study, by observing CD31^+^/NG2^+^ cell ratios, infiltration of M1 cells (especially in the pCR group), and perivascular CD4^+^ T cells (especially in the pCR group), we found that the normalization of vessels was significantly increased in patients treated with this regimen. Considering that normalized vessels can improve the perfusion of blood within the tumour tissue, which in turn brings more oxygen, nutrients, and infiltration of antitumour immune cells to the tumour tissue,^24,36^ and that all of the above factors favour the success of immunotherapy,^37,38^ we believe that the increase in the rate of pCR and MPR in our study is most likely due to the addition of the extra anti-angiogenic drug anilotinib.

In the current trial, the patients had a safety profile consistent with that of individual drugs, and no new safety concerns emerged. The majority of TRAEs were associated with chemotherapy. Only two patients developed cavity formation, and complications like haemoptysis were not observed throughout the treatment. Grade 3 or 4 TRAEs were manageable, and no grade 5 TRAEs were reported. Thus, this therapeutic regimen exhibits both safety and significant improvements in efficacy.

Our study has several limitations. Given the small number of patients enrolled, this study had limited statistical power. As a single-arm study, there was no control arm for comparison, unlike in the AEGAEN trial. The trial design did not allow direct analysis of the relative contributions of perioperative sintilimab and neoadjuvant anlotinib. The postoperative follow-up was also short, preventing analysis of long-term outcomes to determine the best predictive biomarkers of response and their correlation with survival outcomes. An improved understanding of these questions would allow us to advance cancer antiangiogenic agents and immunotherapies. In addition, the trial included only three patients with *EGFR* mutations and excluded patients with *ALK* translocations, so the study findings might not offer any insights into patients with *EGFR* mutations or *ALK* translocations in their tumours, who are known to have a limited response to immunotherapy.^39^

In conclusion, we demonstrate the promising antitumour effects of perioperative immunotherapy with neoadjuvant antiangiogenic therapy in patients with resectable NSCLC. Such a combinatorial therapeutic approach followed by surgical resection and adjuvant sintilimab therapy could offer a new treatment option for patients with resectable NSCLC and should be further explored in advanced trials.

## Materials and methods

### Study design and participants

This open-label, single-arm, phase 2 trial took place at the Department of Thoracic Surgery, Tangdu Hospital, Air Force Medical University, Xian, China, and the Department of Thoracic Surgery, Third Affiliated Hospital of Chongqing Medical University, Chongqing, China. Eligible participants were adults aged 18–75 with histologically confirmed resectable NSCLC (stage IIA-IIIB per AJCC 8th edition). Patients needed to have adequate organ function, an ECOG performance status score of 0 or 1, and at least one measurable lesion according to RECIST version 1.1.^40^ Patients should be deemed to have adequate lung function for resection. Pretreatment tumour tissues should be available for immunohistochemical assessment of PD-L1 expression with the use of the SP263 PD-L1 immunohistochemistry (Ventana Medical Systems, Tucson, AZ, USA). All patients with lung adenocarcinoma underwent testing for genetic alterations, and patients with known *ALK* translocations and who were ROS1 fusion-positive were excluded. The patients could have received no prior chemotherapy or any antitumour therapy, including anti-PD-1 or PD-L1, anti-cytotoxic T-lymphocyte antigen 4 (CTLA-4) treatment, and antiangiogenic therapy. Other key exclusion criteria were active infection requiring systemic therapy, uncontrolled hypertension, active diverticulitis, abdominal abscess, fistulation or perforation, gastrointestinal obstruction, or ongoing haemoptysis (>50 mL/day). The full eligibility criteria are detailed in the study protocol. The study protocol received approval from the Ethics Committees of Tangdu Hospital and the Third Affiliated Hospital of Chongqing Medical University. It adhered to the Declaration of Helsinki and ICH-GCP guidelines. All participants provided written informed consent prior to enrollment, and the study followed CONSORT reporting guidelines. The trial is registered with ClinicalTrials.gov under NCT05400070.

### Treatments

Ten milligrams of anlotinib (Chia Tai Tianqing Pharmaceutical Group Co.) was given orally once on days 1 to 14, and 200 mg of sintilimab (Innovent [Suzhou] Biopharmaceutical Co. Ltd., China) was given intravenously once per three-week cycle on day 1 for a total of three cycles. The patients also received concurrent SOC neoadjuvant platinum-based doublet chemotherapy. Surgery was performed according to local standards within 4–6 weeks of the final dose of neoadjuvant sintilimab and anlotinib plus chemotherapy. The patients were given 200 mg sintilimab intravenously once every 3 weeks for up to one year no later than 6 weeks following surgery. Anlotinib was discontinued if patients developed haemoptysis. For other grade ≥3 treatment-related adverse events (TRAEs), anlotinib was reduced to 8 mg, and the dose of chemotherapy was reduced by 25.0% of the last dose. Dose modification of sintilimab was prohibited. Patients experiencing intolerable adverse events that led to a delay or discontinuation of one drug continued treatment with the remaining study drug. Treatment continued until intolerable toxicity occurred or consent was withdrawn. Those who developed progressive disease were permitted to undergo surgery or receive SOC-CRT at the investigator’s discretion.

### Assessment

Patients underwent radiological evaluations by investigators according to RECIST version 1.1 at baseline, after two cycles of neoadjuvant therapy, prior to surgical resection, and every four cycles during adjuvant therapy until disease progression, intolerable toxicity, death, or withdrawal of consent. Complete and partial responses were confirmed radiologically at least 4 weeks later, while stable disease was confirmed at least 8 weeks after the initial assessment. The ORR was defined as the proportion of patients achieving either a complete or partial response. Patients were followed up by visits or phone calls every 12 weeks to determine survival status.

For pathological assessments, primary lung tumors and lymph node specimens were staged using the AJCC criteria (8th edition). Residual viable tumor percentage in primary tumors was determined from routine hematoxylin and eosin-stained specimens.^32,33^ Tumors with ≤10% viable cells were classified as having MPR, while those with no residual tumor cells (ypT0N0M0) were classified as having pCR. Pathological downstaging was defined as a reduction in ypTNM stage post-treatment, with no new lesions or progression of existing lesions.

### Safety assessments

AEs, including serious AEs (SAEs) and AEs of special interest (AESIs), and laboratory abnormalities were evaluated throughout the treatment period and 30 days after the final dose (up to 90 days for SAEs in the absence of new antitumour therapy) using the Common Toxicity Standards of the National Cancer Institute (NCI CTCAE) version 5.0. The AESIs consisted of immune-related AEs (irAEs), mainly immune-related pneumonitis, myocarditis, and endocrine diseases, and antiangiogenic treatment-related AEs, mainly hypertension, bleeding, and proteinuria.

Postoperative complications were evaluated using the Clavien–Dindo Classification of Surgical Complications. We decided that a treatment was not safe if any grade 5 adverse events occurred.

### End points

The primary end point was pCR. The secondary end points included MPR and EFS. EFS was defined as the interval from enrolment to the earliest occurrence of local progression resulting in inoperability; unresectable tumour, disease progression or recurrence according to RECIST version 1.1 as assessed by the investigator; or death from any cause. OS was defined as the time from enrolment to death from any cause.

### Biomarkers

We utilized CD31^+^ cells to represent endothelial cells, NG2^+^ cells to represent pericytes and the CD31^+^/NG2^+^ cell ratio to reflect vascular normalization.^25^ We calculated the relative CD31^+^ area in each image, defined as the CD31^+^ area divided by the total imaged area. The numbers of CD8^+^ or CD4^+^ T cells within a 100 μm radius from a CD31^+^ structure were calculated to represent perivascular T cells.^41^ We defined Foxp3^+^CD4^+^ T cells as Treg cells,^21,22^ CD11c^+^CD80^+^ cells as M1 macrophages, and CD80^+^CD206^+^ cells as M2 macrophages.^42^ We also calculated the number of VEGF^+^ cells,^43^ PD-1^+^CD8^+^ cells,^27^ and CD39^+^CD8^+^ cells.^44^

### Multiplex immunohistochemical

The paraffin-embedded sections were deparaffinized and rehydrated. After antigen repair, the slides were sealed with hydrogen peroxide and blocked with 10% serum. Subsequently, the slides were incubated with the primary antibodies overnight and incubated with different fluorophore labeled secondary antibodies and the corresponding TSA the next day. After nuclei restained with DAPI, quenching tissue autofluorescence and sealing tablets, panoramic scans of the slides were performed (Nikon Eclipse C1, Pannoramic MIDI). Antibodies against the following proteins were used for the study: CD31 (GB11063-1), CD4 (ab133616), CD8 (GB12068), FOXP3 (GB112325), CD80 (GB114055), CD11c (GB11059), CD206 (24595T), CD39 (GB111582), PD-1 (GB12338), NG2 (GB111915).

### Quantification of staining data

The panoramic scans images of slides were acquired as described above. The Indica Labs-HighPlex module of Halo software was used to calculate the number of single-positive cells and double-positive cells in the target area of each section (servicebio Technology CO., LTD, Wuhan), such as VEGF^+^ cells, CD4^+^Foxp3^+^ double-positive cells. The Indica Labs-Area Quantification module of Halo software was used to quantify the area of positive cells (servicebio Technology CO., LTD, Wuhan). For example, CD31^+^ areas was calculated as mentioned above and CD4^+^ or CD8^+^ cells within a 100 μm radius from the CD31^+^ area were calculated with the Spatial Analysis module and Indica Labs-HighPlex module of Halo software (servicebio Technology CO., LTD, Wuhan). For each CD31^+^ segment, numbers of CD8^+^ or CD4^+^ T cells within the defined area were normalized by the area of the CD31^+^ segment.

### Statistical analysis

The sample size calculation used Simon’s two-stage maximum value design. Based on the Checkmate-816 trial,^5^ the minimum pCR rate was set at 25%, and the expected pCR rate was 45%. Assuming a type I error rate (α) of 0.05 and a type II error rate (β) of 80%, a sample size of 41 patients was needed. Seventeen patients were enrolled in stage 1. If more than five patients achieved pCR, the trial proceeded to stage 2; otherwise, the trial was terminated.

All the statistical analyses were prespecified and were conducted using SAS version 9.4. The study followed the ITT principle, and efficacy analyses were based on the ITT population, which included all the enrolled patients. The PP set included all patients who received surgery and had at least one after treatment radiological evaluation with no major protocol violations. Patients were censored at the final follow-up visit if they did not have an event. EFS and OS were calculated utilizing the Kaplan–Meier method. The safety set comprised all patients who received at least one dose of sintilimab or anlotinib in combination with chemotherapy during the neoadjuvant treatment phase, as well as those who received at least one dose of sintilimab during this period. Safety data were mainly analyzed through descriptive statistics.

## Supplementary information

Sigtrans_Supplementary_Materials_Word_template

Study protocol

## Data Availability

The data supporting the findings of this study could be available for scientific purpose from the corresponding author (yanxiaolong@fmmu.edu.cn) 24 months after study completion.
