# Supplementary material for: Perioperative sintilimab and neoadjuvant anlotinib plus chemotherapy for resectable non-small-cell lung cancer: a multicentre, open-label, single-arm, phase 2 trial (TD-NeoFOUR trial)
Source: Signal Transduct Target Ther. 2024 Oct 28;9:296. doi: 10.1038/s41392-024-01992-0 (PMC11514280; doi:10.1038/s41392-024-01992-0)
Supplement: Supplementary file 1 — Sigtrans_Supplementary_Materials_Word_template [file 41392_2024_1992_MOESM1_ESM.docx]

Supplementary Materials for

Perioperative sintilimab and neoadjuvant anlotinib plus chemotherapy for resectable non-small-cell lung cancer: a multicentre, open-label, single-arm, phase 2 trial

Hongtao Duan^1,╋^, Changjian Shao^1,╋^, Zhilin Luo^2,╋^, Tianhu Wang^2,╋^, Liping Tong^1,╋^, Honggang Liu^1^, Xin Yao^1^, Jie Lei^1^, Jinbo Zhao^1^, Yuan Gao^3,*^, Tao Jiang^1,*^, Xiaolong Yan^1,*^

Correspondence to: [yanxiaolong@fmmu.edu.cn](mailto:yanxiaolong@fmmu.edu.cn), [jiangtaochest@163.com](mailto:jiangtaochest@163.com), gaoyuan321@fmmu.edu.cn

**This PDF file includes:**

Supplementary Text

Figures. S1 to S8

Tables S1 to S3

Supplementary Text

**Specific circumstances of patients**

Since the patients (p12, p20, p27, p28, p29, p30, p33, and p34) or their family members refused further treatment after 2 cycles of neoadjuvant therapy before surgery, surgical treatment was chosen.

Patients p8 and p22 stopped receiving anlotinib due to bronchopulmonary haemorrhage after the second cycle, and haemostatic treatment stopped the haemorrhage had been suspend. Anlotinib treatment was not continued in the third cycle.

Patient p41 developed pneumonitis after the second cycle, which improved after receiving hormone treatment. In the third cycle, sintilimab was not included.

Nine patients did not complete the adjuvant treatment protocol after surgery.

Patients p9, p10, and p14 had adenocarcinoma, and next-generation sequencing (NGS) revealed EGFR 19 del and L858 mutations. Postoperative pathology showed that the patients did not reach MPR. No adjuvant treatment with sintilimab but rather targeted treatment was given.

Patient p2 experienced a grade 2 decrease in cortisol function postoperatively and at the termination of subsequent treatment

Patient p6 underwent surgery, and there was fatigue of grade 3 or above. Adjuvant treatment was refused.

Patient p15 refused adjuvant therapy after surgery.

After patient p23 received 3 cycles of adjuvant sintilimab therapy, grade 2 immune pneumonitis developed, and further treatment was discontinued

Patient p24: There was a decrease in cortisol function above grade 3 postoperatively, and no treatment was given.

Patient p25: After adjuvant treatment with 6 cycles of sintilimab, a decrease in the cortisol concentration of grade 3 or above was detected, and treatment was stopped.

Figure. S1


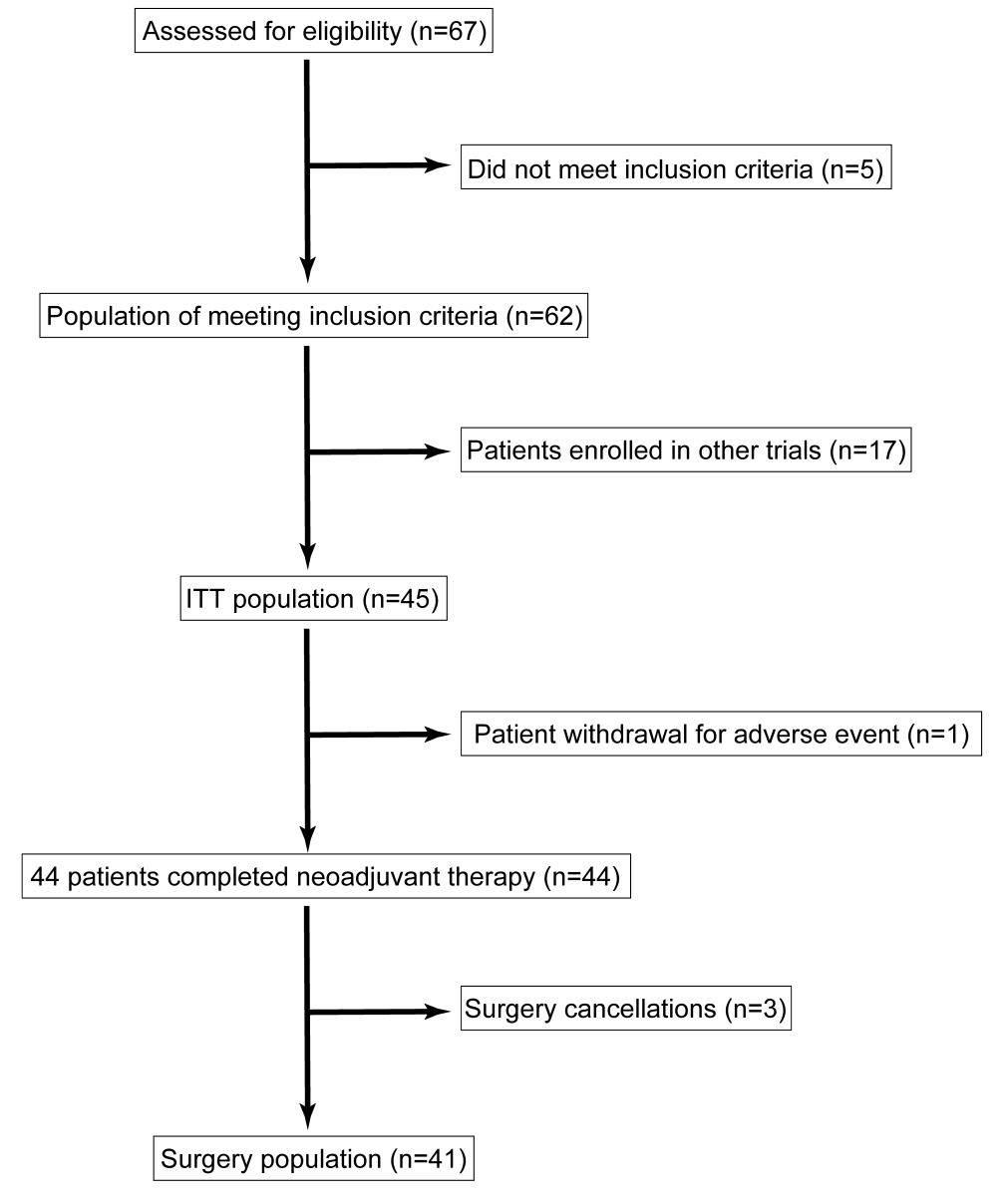


Figure. S1. The study flowchart.

Figure. S2


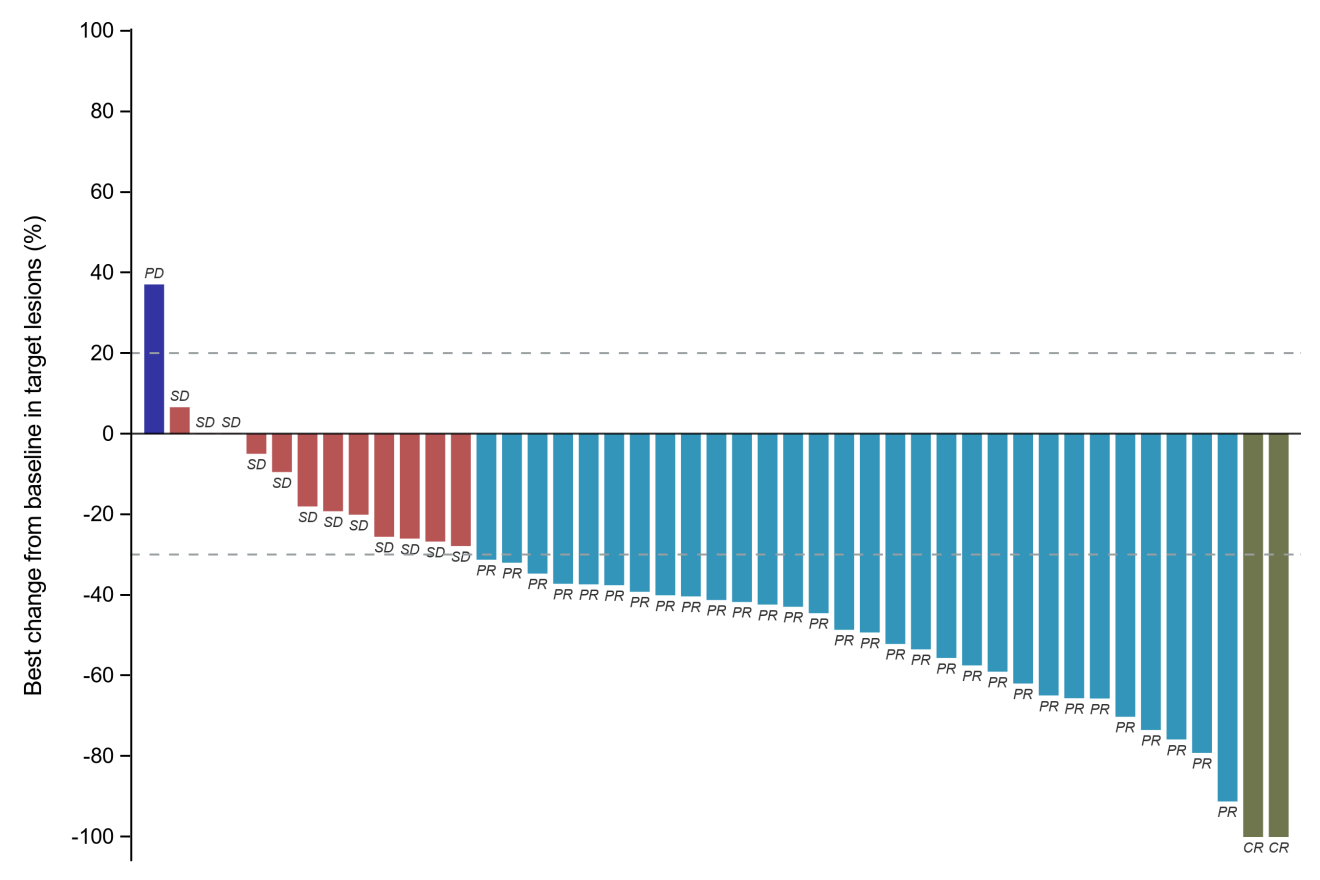


Figure. S2. Treatment response. Waterfall plots of the best percentage changes from baseline for the sum of target lesion diameters are shown for individual patients. Each bar represents one patient in the intention-to-treat population. Dashed horizontal lines indicate a 30% reduction or a 20% increase in target lesion size. Percentage changes greater than 100% are truncated at 100%.

Figure. S3


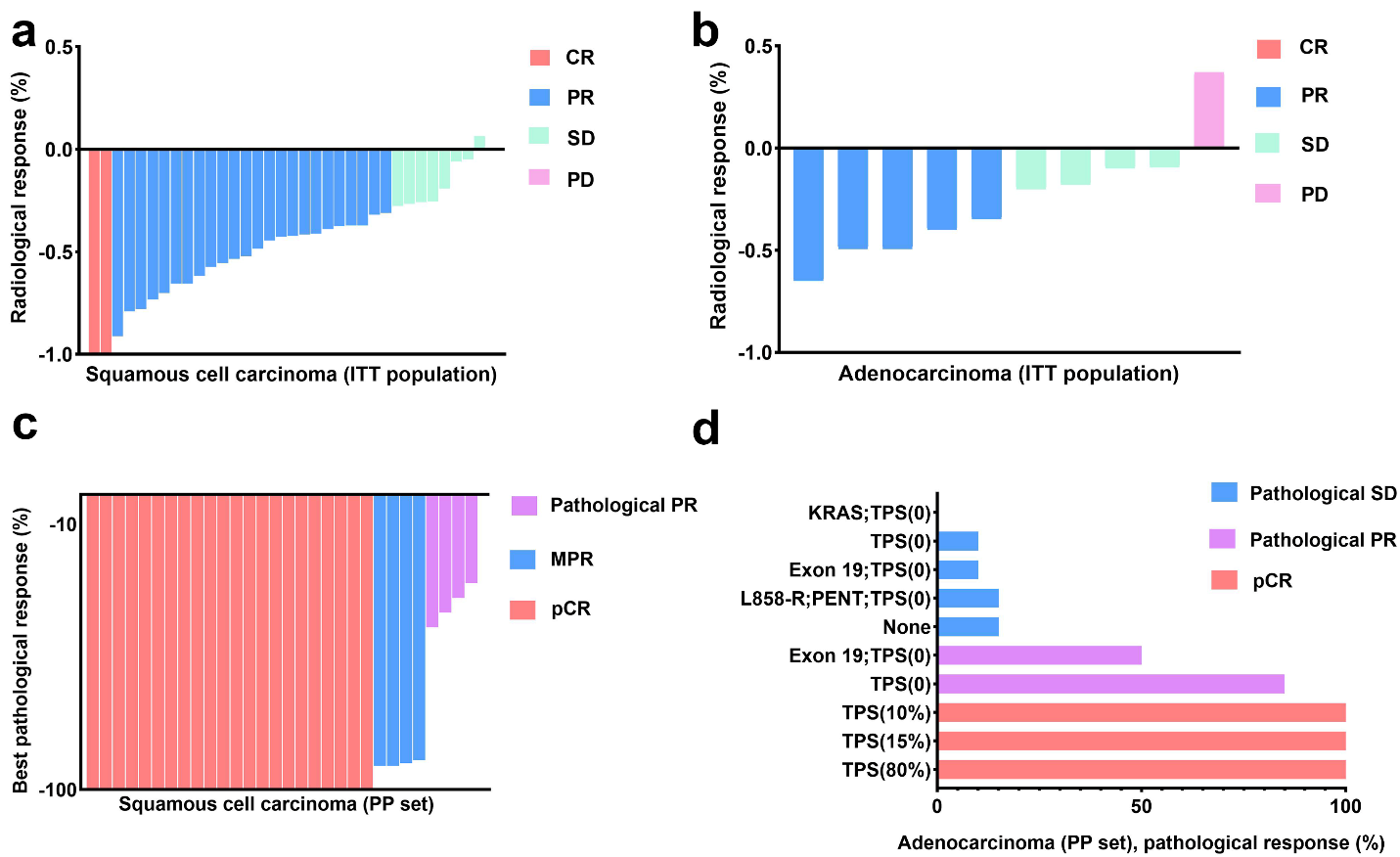


Figure. S3. Waterfall plots of the radiological response and best pathological response of patients with squamous cell carcinoma (a, c) and adenocarcinoma (b, d). Each bar represents one patient in the intention-to-treat population (a, b) and per-protocol set (c, d). CR, complete response. PR, partial response. SD, stable disease. PD, progressive disease. pCR, pathological complete response. MPR, major pathological response. Pathological PR, pathological partial response. Pathological SD, pathological stable disease.

Figure. S4


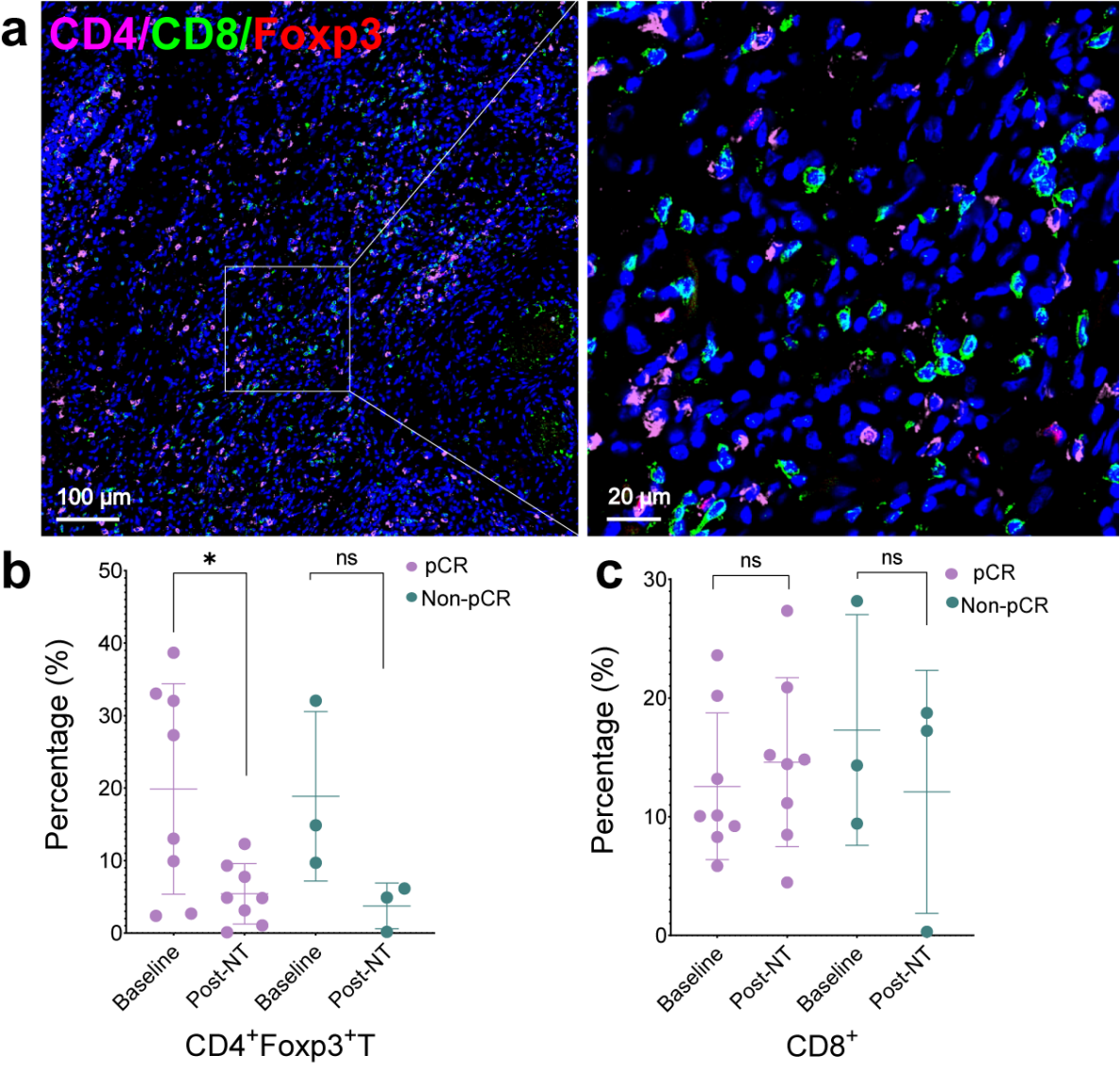


**Figure. S4.** **Representative images and quantification of multiplex immunohistochemical staining of tumours at baseline and after neoadjuvant treatment (post-NT).** (a) CD4^+^ Foxp3^+^ T cell infiltration and CD8^+^ T cell infiltration in the tumor microenvironment was assessed before and after treatment, while CD4 staining (pink), Foxp3 staining (red) and CD8 staining (green) indicates the infiltration of corresponding immune cells. (b) Statistical analysis of the infiltration of CD4^+^ Foxp3^+^ T cell was performed in baseline and post-neoadjuvant treatment samples within the pCR group (paired samples, n=8) and the non-pCR group (paired samples, n=3). (c) Statistical analysis of the infiltration of CD8^+^ T cell was performed in baseline and post-neoadjuvant treatment samples within the pCR group (paired samples, n=8) and the non-pCR group (paired samples, n=3). Data are mean ***±*** SD. (b-c) Wilcoxon paired *t* test. No Significance (ns), ns: p > 0.05, *p < 0.05, and **p < 0.01.

Figure. S5


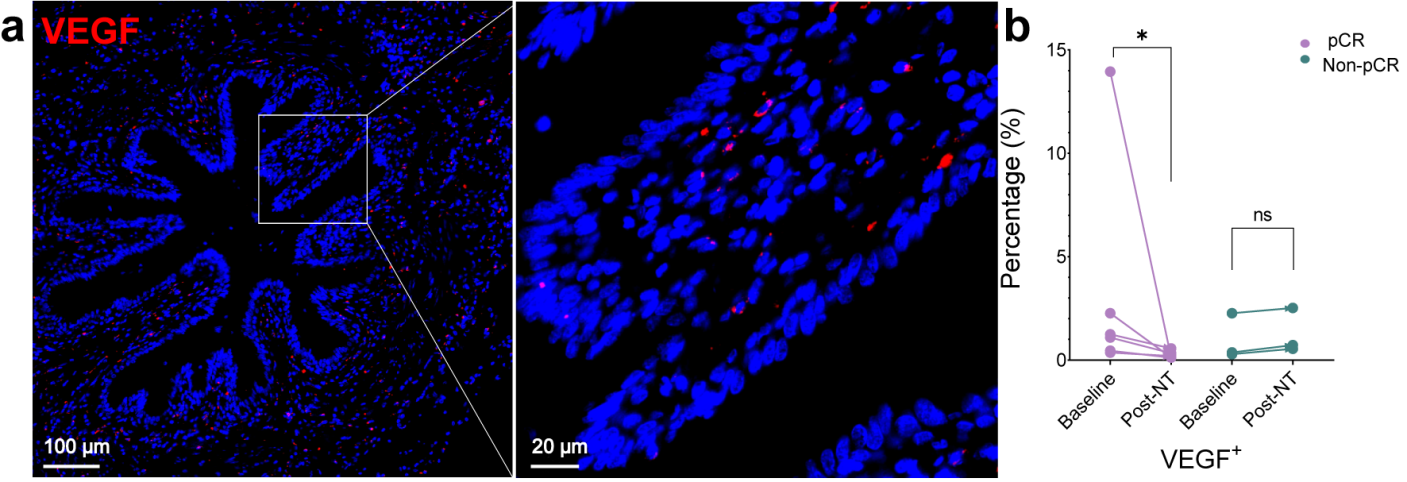


**Figure. S5.** **Representative images and quantification of multiplex immunohistochemical staining of tumours at baseline and after neoadjuvant treatment (post-NT).** (a) VEGF^+^ cell (staining red) infiltration in the tumor microenvironment was assessed before and after treatment. (b) Statistical analysis of the infiltration of VEGF^+^ cell was performed in baseline and post-neoadjuvant treatment samples within the pCR group (paired samples, n=6) and the non-pCR group (paired samples, n=3). Data are mean ***±*** SD. (b) Wilcoxon paired *t* test. No Significance (ns), ns: p > 0.05, *p < 0.05, and **p < 0.01.

Figure. S6


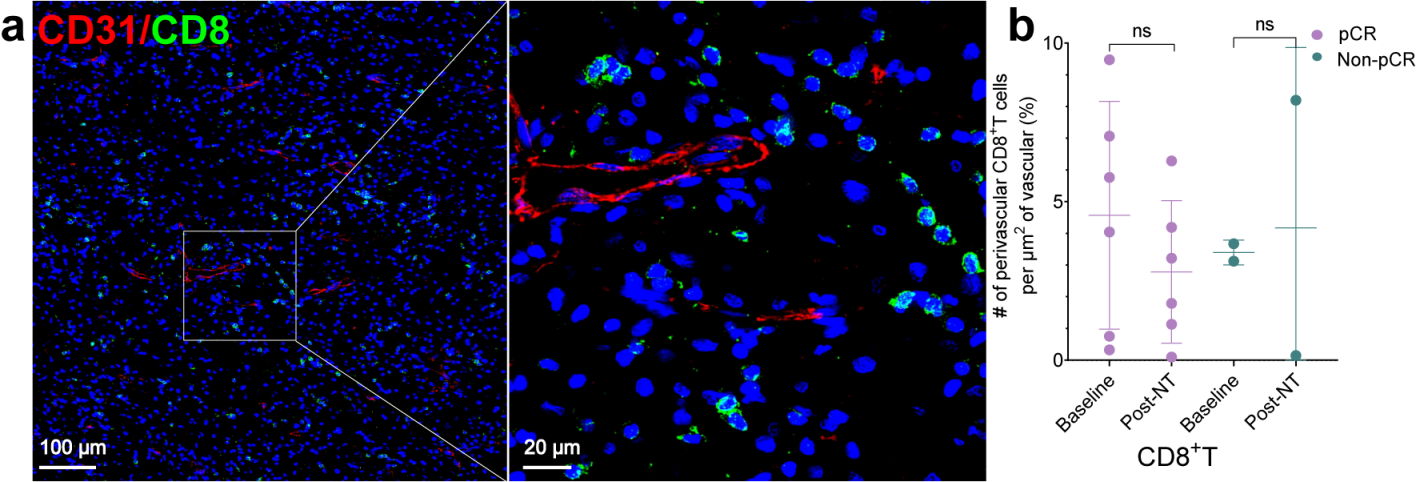


**Figure. S6.** **Representative images and quantification of multiplex immunohistochemical staining of tumours at baseline and after neoadjuvant treatment (post-NT).** (a) Perivascular CD8^+^ T cells per μm² of vascular area. CD31 staining (red) represents the vascular area, while CD8 staining (green) indicates the infiltration of corresponding immune cells. (b) Statistical analysis of perivascular CD8^+^ T cell count per μm² of vascular area in baseline and post-neoadjuvant treatment samples within the pCR group (paired samples, n=6) and the non-pCR group (paired samples, n=2). Data are mean ***±*** SD. (b) Wilcoxon paired *t* test. No Significance (ns), ns: p > 0.05, *p < 0.05, and **p < 0.01.

Figure. S7


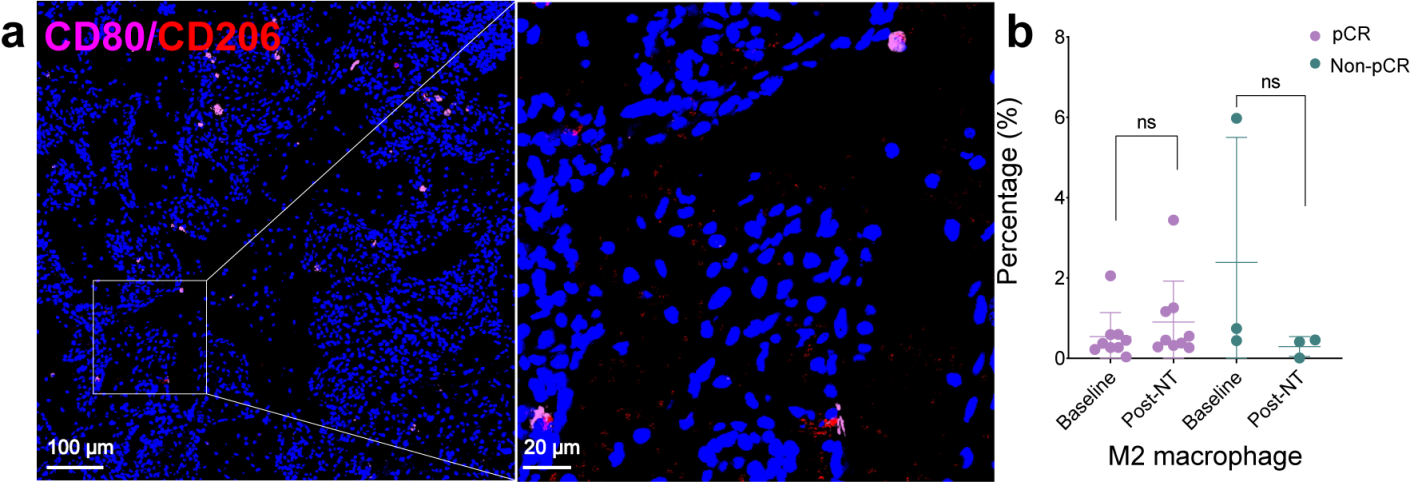


**Figure. S7. Representative images and quantification of multiplex immunohistochemical staining of tumours at baseline and after neoadjuvant treatment (post-NT).** (a) Assessment of the expression levels of CD80 (pink) and CD206 (red) in the lung cancer microenvironment before and after treatment, along with statistical analysis of the infiltration of CD206^+^CD80^+^ M2 macrophage. (b) Statistical analysis of the CD206^+^CD80^+^ M2 macrophage in baseline and post-neoadjuvant treatment samples within the pCR group (paired samples, n=9) and the non-pCR group (paired samples, n=3). Data are mean ***±*** SD. (b) Wilcoxon paired *t* test. No Significance (ns), ns: p > 0.05, *p < 0.05, and **p < 0.01.

Figure. S8


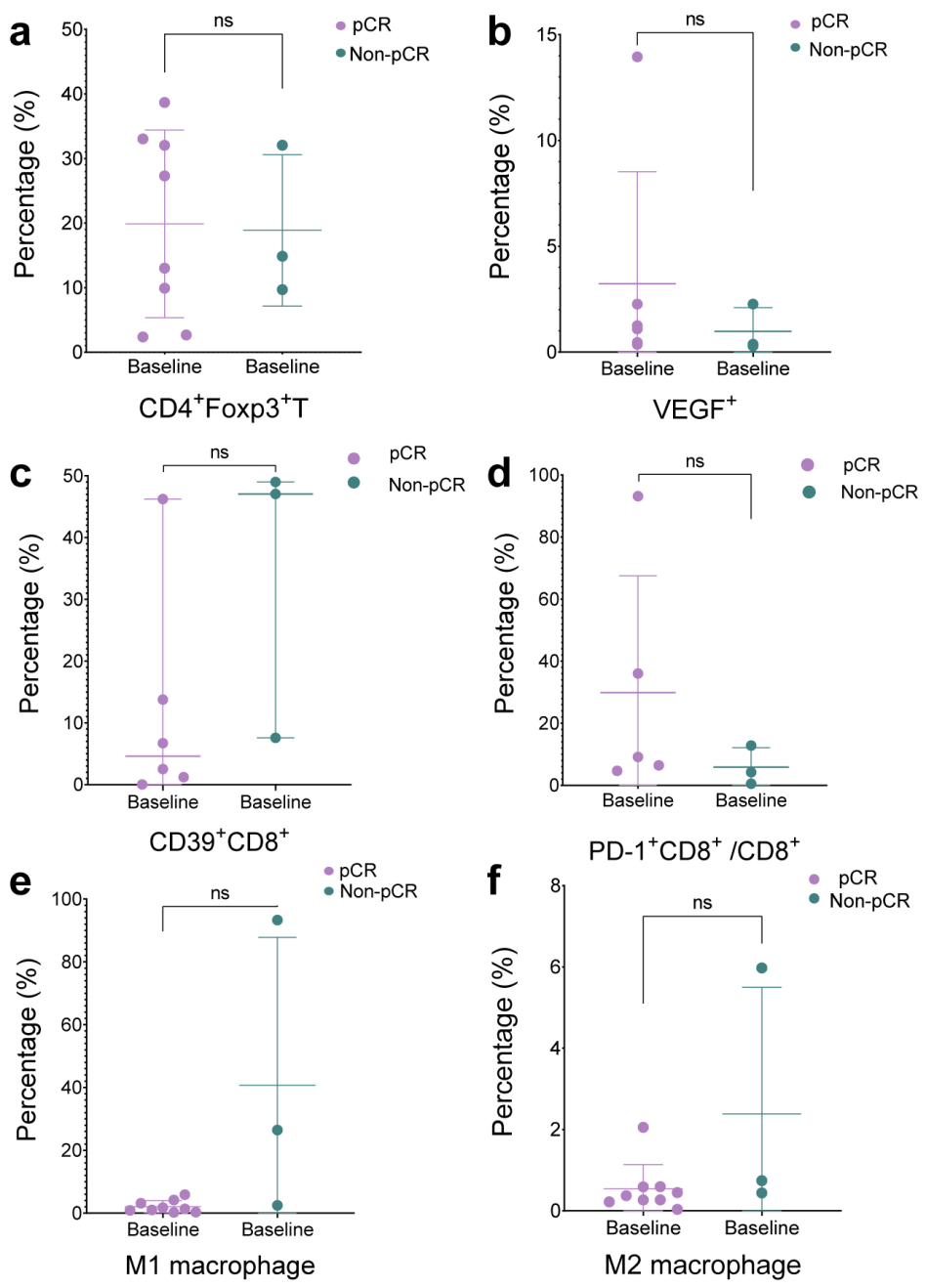


**Figure. S8. Statistical analysis of multiplex immunohistochemical staining of tumours at baseline.** (a) Statistical analysis of CD4^+^Foxp3^+^ T cell infiltration within baseline samples (pCR group, n=8; non-pCR group, n=3). (b) Statistical analysis of frequency of VEGF-positive cells within baseline samples (pCR group, n=6; non-pCR group, n=3). (c) Statistical analysis of frequency of CD39^+^CD8^+^ T cells within baseline samples (pCR group, n=6; non-pCR group, n=3). (d) Statistical analysis of frequency of PD-1^+^CD8^+^ cells among CD8^+^ T cells within baseline samples (pCR group, n=5; non-pCR group, n=3). (e) Statistical analysis of frequency of M1 macrophages within baseline samples (pCR group, n=9; non-pCR group, n=3). (f) Statistical analysis of frequency of M2 macrophages within baseline samples (pCR group, n=9; non-pCR group, n=3). Data are mean ***±*** SD. (a-f) Wilcoxon unpaired *t* test. No Significance (ns), ns: p > 0.05, *p < 0.05, and **p < 0.01.

Table S1. Surgical characteristics of the PP set (N=41).

| Variable | Value |
| --- | --- |
| Extent of resection |  |
| R0 | 38 (93) |
| R1 | 3 (7) |
| Median operative time (range), min | 170 (40-325) |
| Median estimated blood loss (range), mL | 100 (10-800) |
| Median postoperative hospital stays (range), days | 8 (3-25) |
| Median chest tube duration (range), days | 8 (2-16) |
| Extent of surgery |  |
| Lobectomy | 27 (66) |
| Sleeve resection/bronchoplasty | 8 (20) |
| Bilobectomy | 3 (7) |
| Pneumonectomy | 2 (5) |
| Biopsy | 1 (2) |
| Surgical methods |  |
| VATS/RATS | 38 (93) |
| Conversion to thoracotomy | 2 (5) |
| Thoracotomy | 1 (2) |

Data are expressed as number (%) unless otherwise specified.

PP: per-protocol; RATS: Robotic-assisted thoracic **surgery;** VATS: **video-assisted thoracoscopic surgery**

Table S2. Analysis in EFS

| Variable | N=45 |
| --- | --- |
| Censored, n (%) | 36 (80.0) |
| Median (95% CI) | NR (25.1, NE) |
| 6 m-EFS, % (95% CI) | 100.0 (100.0, 100.0) |
| 12 m-EFS, % (95% CI) | 97.7 (84.6, 99.7) |
| 24 m-EFS, % (95% CI) | 81.5 (64.5, 90.9) |

Table S3. *Post hoc* subgroup analysis in EFS

| Variable | N | Event | Median (95% CI) | P |
| --- | --- | --- | --- | --- |
| Sex |  |  |  |  |
| Male | 41 | 8 | NR (25.1, NE) | 0.9714 |
| Female | 4 | 1 | NR (22.2, NE) |  |
| Age |  |  |  |  |
| < 60 | 17 | 3 | NR (22.2, NE) | 0.5864 |
| ≥ 60 | 28 | 6 | NR (25.1, NE) |  |
| Smoking |  |  |  |  |
| Nonsmoker | 8 | 1 | NR (22.2, NE) | 0.5845 |
| Former smoker | 9 | 3 | NR (18.6, NE) |  |
| Current smoker | 28 | 5 | NR (24.3, NE) |  |
| ECOG |  |  |  |  |
| 0 | 34 | 9 | NR (24.3, NE) | 0.1311 |
| 1 | 11 | 0 | NR (NE, NE) |  |
| Histological status |  |  |  |  |
| Squamous cell carcinoma | 34 | 6 | NR (25.1, NE) | 0.8225 |
| Adenocarcinoma | 10 | 3 | NR (18.9, NE) |  |
| Sarcomatoid carcinoma | 1 | 0 | NR (NE, NE) |  |
| Clinical stage |  |  |  |  |
| IIA | 4 | 0 | NR (NE, NE) | 0.3160 |
| IIB | 8 | 1 | NR (18.9, NE) |  |
| IIIA | 15 | 2 | NR (24.3, NE) |  |
| IIIB | 18 | 6 | NR (22.21, NE) |  |
| Tumour stage |  |  |  |  |
| T1 | 4 | 0 | NR (NE, NE) | 0.6250 |
| T2 | 8 | 1 | NR (18.69, NE) |  |
| T3 | 16 | 4 | NR (22.21, NE) |  |
| T4 | 17 | 4 | NR (16.46, NE) |  |
| Node stage |  |  |  |  |
| N0 | 11 | 1 | NR (13.08, NE) | 0.6160 |
| N1 | 10 | 2 | NR (18.69, NE) |  |
| N2 | 24 | 6 | NR (22.21, NE) |  |
