# Supplementary material for: Perioperative sintilimab and neoadjuvant anlotinib plus chemotherapy for resectable non-small-cell lung cancer: a multicentre, open-label, single-arm, phase 2 trial (TD-NeoFOUR trial)
Source: Signal Transduct Target Ther. 2024 Oct 28;9:296. doi: 10.1038/s41392-024-01992-0 (PMC11514280; doi:10.1038/s41392-024-01992-0)
Supplement: Supplementary file 2 — Study protocol [file 41392_2024_1992_MOESM2_ESM.pdf]

A Prospective Single-arm, Open-label Study of Sintilimab and Anlotinib Combined with Chemotherapy in Neoadjuvant Treatment of Resectable NSCLC

Principal Investigator: Prof. Xiaolong Yan

Leading Site: Tangdu Hospital, Air Force Medical University

Study Period: March 2021 - December 2026

Version No.: 2.1

Version Date: March 17th, 2021

Content

Program Overview 4

Study Flow Chart 10

List of Abbreviations 14

1. Research background and rational for the study 16

1.1 Research background 16

1.1.1 Lung cancer and its neoadjuvant therapy 16

1.1.2 Research on molecular markers related to angiogenesis in NSCLC 17

1.1.3 Neoadjuvant studies of NSCLC and PD-1 inhibitors 18

1.2 Rational for the study 18

2. Research objectives 19

3. Study Design 20

3.1 Description of research design 20

3.2 Primary endpoints 20

3.3 Secondary endpoints 20

3.4 Sample size 20

3.5 Inclusion and exclusion criteria 20

3.5.1 Inclusion criteria 20

3.5.2 Exclusion criteria 22

3.5.3 drop-out/eliminate criteria 23

3.5.4 Termination criteria 23

3.6 Treatment management 24

3.6.1 Treatment plan 24

3.6.2 Dose adjustment and discontinuation of administration 26

3.6.3 Drug management 26

3.6.4 Treatment prescription 26

3.7 Duration of research 26

4. Research procedures and data collection 27

4.1 Data collection program 27

4.2 Screening phase 27

4.3 Preoperative treatment period 28
4.4 Radical surgery 29
4.5 Follow-up period 29
4.6 Additional follow-up 29
4.7 Survival visit 30
4.8 Adverse event follow-up 30
4.9 Handling steps for patient withdrawal from follow-up program 30
5. Statistical methods. 30
5.1 Analysis variables 30
5.2 Statistical methods. 32
5.3 Determination of sample size 33
6. Adverse event reporting 33
6.1 Adverse event observation 33
6.2 AE rating 34
6.3 AE record 34
6.4 Determination of the relationship between AEs and the test drug 35
6.5 SAE 35
6.6 irAE 37
6.7 Postoperative complication 38
7. Quality control and quality assurance 38
8. Ethical, regulatory and administrative principles 39
8.1 Ethical principles 39
8.2 Laws and regulations 39
8.3 Data protection 39
8.4 Confidentiality agreement 39
9. Revised program 39
10. Reference 39

Program Overview

Research Title	A Prospective Single-arm, Open-label Study of Sintilimab and Anlotinib Combined with Chemotherapy in Neoadjuvant Treatment of Resectable NSCLC
Research Objects	Patients with Operable Stage IIA, IIB, IIIA, IIIB NSCLC
Applicant	Initiated by investigators
Principal investigators	Prof. Xiaolong Yan
Research Organization	Tangdu Hospital, Air Force Medical University
Research Objective	To evaluate the efficacy and safety of neoadjuvant therapy for IIA-IIIB NSCLC by sintilimab and anlotinib in combination with chemotherapy.

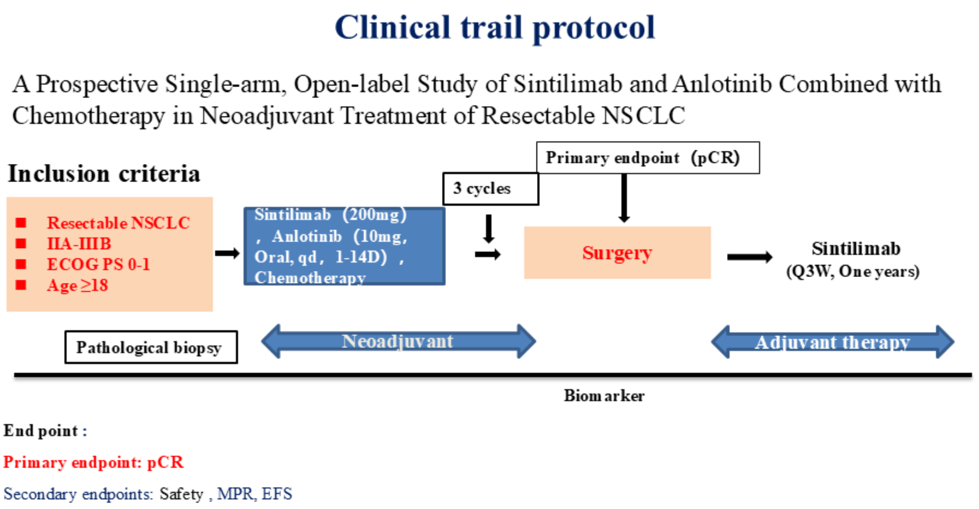

Planned No. of Patients to be Enrolled	45 cases
Patient Selection Criteria	**Inclusion Criteria:**<br>Subjects must meet the following inclusion criteria:<br>1) Age ≥ 18 and ≤ 75 years with a ECOG PS score of 0-1;<br>2) Subjects who can adhere to the study protocol;<br>3) At least one measurable lesion according to RECIST 1.1

criteria;

4) Stage IIA, IIB, IIIA, and IIIB NSCLC with histologically or cytologically confirmed resectable NSCLC according to International Association for the Study of Lung Cancer and the 8th edition lung cancer TNM staging of American Joint Committee on Cancer Staging;

5) Participants must have a tumor tissue sample available for PD-L1 immunohistochemistry (IHC) and must submit a formalin-fixed, paraffin-embedded (FFPE) tissue block or unstained tumor tissue section and corresponding pathology report prior to enrollment. Tissue must be biopsy specimens from hollow-needle aspiration biopsies, excisions, or incisions. Fine-needle aspiration biopsies obtained by EBUS are insufficient for biomarker review and as a basis for randomization;

6) Tumor tissue samples must be fresh or obtained within 3 months prior to enrollment;

7) For all suspicious mediastinal lymph nodes evaluated by PET-CT or contrast CT findings, a pathological diagnosis was not necessarily required;

8) Good hematopoietic function, defined as absolute neutrophil count $\geq 1.5\times10^9$/L (not receiving granulocyte colony-stimulating factor supportive therapy), platelet count $\geq 100\times10^9$/L, hemoglobin ≥ 90 g/L; (no transfusion or no erythropoietin dependence within 7 days);

9) Good liver function, defined as serum total bilirubin ≤ 1.5 times the upper limit of normal (ULN); glutamic oxaloacetic transaminase (AST) and glutamic pyruvic transaminase (ALT) levels ≤ 2.5 times ULN in patients without hepatic metastases; and AST and ALT levels ≤ 5 times ULN in patients with documented hepatic metastases;

10) Good renal function, defined as serum creatinine ≤1.5 times ULN or calculated creatinine clearance ≥ 60 ml/min (Cockcroft-

Gault formula); urine protein less than 2+ on routine urinalysis, or 24-h urine protein <1 g;

11) Good coagulation function, defined as an international normalized ratio (INR) or prothrombin time (PT) ≤ 1.5 times ULN;

12) Total lung function (e.g., FVC, FEV1, TLC, FRC, and DLco) capable of tolerating the proposed lung resection, as assessed by the surgeon;

13) For female subjects of childbearing potential, a negative urine or serum pregnancy test should be performed within 3 days prior to receiving the first dose (Cycle 1, Day 1);

14) If there is a risk of conception, male and female patients are required to use highly effective contraception and continue until at least 180 days after discontinuation of treatment.

Exclusion Criteria:

1) Presence of locally advanced unresectable or metastatic disease;

2) Participants with known ROS1 fusion-positive or ALK translocations;

3) ≥ grade 2 peripheral neuropathy;

4) Active autoimmune diseases (known or suspected). Type I diabetes, hypothyroidism requiring only hormone replacement therapy, skin disorders that do not require systemic therapy (e.g., vitiligo, psoriasis, or alopecia areata), or disorders that are not expected to recur in the absence of external stimuli are eligible for enrollment;

5) Those requiring systemic treatment with glucocorticoids (>10 mg prednisone equivalent dose per day) or other immunosuppressive drugs within 14 days prior to randomization. Inhaled or topical steroids are permitted in the absence of active autoimmune disease;

6) Active hepatitis B/hepatitis C infection and known HIV

	infection;<br>7) Patients with prior chemotherapy or any other anti-tumor therapy;<br>8) Patients having received the following therapies in the past: anti-PD-1, anti-PD-L1, anti-PD-L2, or anti-CTLA-4 antibodies or any other antibody targeting T-cell co-regulatory pathways;<br>9) Patients who have been treated with anti-angiogenic drugs, such as anlotinib;<br>10) Patients with the presence of uncontrollable hypertension;<br>11) Presence of clinically uncontrollable pleural effusion or abdominal effusion;<br>12) Presence of clinically active diverticulitis, abdominal abscess, gastrointestinal obstruction;<br>13) Have received a solid organ or blood system transplant;<br>14) Diagnosis of other malignancies within 5 years prior to the first dose, excluding carcinoma that have been apparently cured, such as basal or squamous cell skin cancer, superficial bladder cancer, or carcinoma in situ of the prostate, cervix, or breast;<br>15) Have active infection requiring systemic therapy;<br>16) Vaccination within 30 days prior to the first dose (Cycle 1, Day 1);<br>17) Patients with hemoptysis (>50 mL/day);<br>18) Patients whose tumors have invaded important blood vessels or who are at risk of hemorrhage during subsequent studies.
Termination Criteria	1) Disease progression;<br>2) Grade 3/4 adverse events failure to complete treatment after dose adjustment according to the trial protocol;<br>3) Death;<br>4) Occurrence of unanticipated and unacceptable adverse drug reactions;<br>5) Delayed dosing for >4 weeks.
Drop-out/Eliminate	Failure to administer the medication in accordance with the

Criteria	dosage, method and course of treatment specified in this research program.<br>Note: Cumulative discontinuation of more than two weeks in a dosing cycle is recorded as drop-out.
Withdrawal Criteria	Patients can withdraw from the study at any time, without giving a reason.<br>Patients must withdraw from the study if any of the following events occur:<br>1) Patient voluntarily withdraws from the study.<br>2) Subject or his/her legal representative requests to withdraw from the study;<br>3) There are medical or ethical reasons affecting the continuation of the study;<br>4) The investigator determines that withdrawal from the study is necessary in the best interest of the subject;<br>5) The subject loses to follow-up;<br>6) Subject pregnancy;<br>7) Serious violation of the trial protocol (including serious violation of study enrollment criteria).
Dosing Regimen	Sintilimab (200mg fixed dose) iv, d1, q3w + anlotinib 10mg, po, qd1-14, q3w + platinum-doublet chemotherapy, evaluated after 3 cycles of combination therapy and discontinued for 3 weeks (21 days) followed by surgery, which was performed within 4-6 weeks (22-42 days) of the last dose.
Key Observation Index	Pathologic Complete Response (PCR) rate, safety evaluation
Secondary Observational Indexes	Major Pathologic Response (MPR) rate, Event-Free Survival (EFS), and Overall Survival (OS)

Other Safety Indexes	Observe any adverse events, including abnormal clinical symptoms and vital signs, abnormalities in laboratory tests, occurring during the clinical study in all subjects, record their clinical manifestations, severity, time of occurrence, duration, management and prognosis, and determine the correlation between them and the test drug. The safety of the study drugs was evaluated by NCI-CTCAE version 5.0 criteria.

Study Flow Chart

treatment cycle / Item	Screening period		Treatment period			7 days before the planned date of surgery	3-6 weeks after surgery	Follow-up period (after completion of treatment)	
	d-14~ d-1	d-7~ d-1	C1	C2	C3			Safety follow-up [19]	Survival follow-up [20]
Informed consent	x								
Demographic information	x								
Past medical and treatment history	x								
Verification of enrollment criteria		X							
ECOG score [1]		X	x	x	x	x	x	x	
Vital signs examination [2]		X	x	x	x	x	x	x	
Physical examination [3]		x	x	x	x	x	x	x	
Virologic index examination [4]	x								
Blood routine [5]		x		x	x	x	x	x	
Urine routine [6]		x		x	x	x	x	x	
Blood biochemistry [7]		x		x	x	x	x	x	
Stool routine [8]		x		x	x	x	x	x	
Coagulation function [9]		x				x	x	x	
Thyroid function [10]		x		x	x	x	x	x	
12-lead electrocardiogram [11]		x		x	x	x	x	x	
Echocardiogram [12]		x							
Pregnancy test [13]		x				x	x	x	
Imaging [14]	x				x	x		x	x
Lung function assessment [15]	x					x			
Pathologic evaluation [16]	x						x		
Adverse events [17]	x	x	x	x	x	x	x	x	x
Concomitant medications [18]	x	x	x	x	x	x	x	x	x
Myocardial enzymes [21]		x		x	x	x	x	x	
Drug of this study [22]			x	x	x				

Note: In addition to the examination items and time points listed in the table, investigators may add visits and other examinations as needed, and the results should be completed in the appropriate section of the case report form (e.g., *Unscheduled Visits and Examinations*); the planned time window for this study is ±7 days unless otherwise noted.

[1] ECOG scores: performed within 7 days prior to first dose, prior to dosing on d1 of each treatment cycle, 7 days prior to surgical resection, 3-6 weeks post-surgery, end of treatment/withdrawal from the study, and at Visit 1 of the safety follow-up period.

[2] Vital signs: pulse, respiratory rate, temperature, and blood pressure; performed within 7 days prior to the first dose, prior to dosing on d1 of each treatment cycle, 7 days prior to surgical resection, 3-6 weeks post-surgery, end of treatment/withdrawal from the study, and at Visit 1 of the safety follow-up period.

[3] Physical examination: comprehensive physical examination (general condition, head, face, skin, lymph nodes, eyes, ear, nose and throat, oral cavity, respiratory system, cardiovascular system, abdomen, genitourinary system, musculoskeletal system, nervous system, and mental status) prior to dosing on day 1 of each treatment cycle and at the end of treatment/withdrawal from the study; performed within 7 days prior to the first dose, prior to dosing on d1 of each treatment cycle, 7 days prior to surgical resection, 3-6 weeks after surgery, at the end of treatment/withdrawal from the study, and at Visit 1 of the safety follow-up period.

[4] Virology test: HBsAg, HBsAb, HBeAg, HBeAb, HBcAb, HBV DNA (qualitative, quantitative test if positive), HCV-Ab (quantitative HCV-RNA test if positive) and HIV-Ab.

[5] Blood routine: red blood cell count (RBC), hemoglobin (Hb), platelet count (PLT), white blood cell count (WBC), neutrophil count (ANC), lymphocyte count; performed within 7 days prior to the first dose, prior to the d1 dose of the second and third treatment cycles, 7 days prior to surgical resection, 3-6 weeks post-surgery, at the end of the treatment/withdrawal from the study, and at Visit 1 of the safety follow-up period.

[6] Urine routine: white blood cells, red blood cells, urine protein. Performed within 7 days prior to the first dose, prior to d1 dosing in the second and third treatment cycles, 7 days prior to surgical resection, 3-6 weeks after surgery, end of treatment/withdrawal from the study, and at Visit 1 of the safety follow-up period.

[7] Blood biochemistry: glutamic pyruvic transaminase (ALT), glutamic oxaloacetic transaminase (AST), glutamyl transpeptidase (γ-GT), total bilirubin (TBIL), direct bilirubin (DBIL), alkaline phosphatase (AKP), blood urea nitrogen (BUN), total protein (TP), albumin (ALB), creatinine (Cr), blood glucose (GLU), K^+, Na^+, Ca^{2+}, Mg^{2+}, Cl^-; performed within 7 days prior to the first dose, prior to dosing on d1 of the second and third treatment cycles, 7 days prior to surgical resection, 3-6 weeks post-surgery, at the end of treatment/withdrawal from the study, and at Visit 1 of the safety follow-up period.

[8] Stool routine: performed within 7 days prior to the first dose, prior to d1 dose in the second and third treatment cycles, 7 days prior to surgical resection, 3-6 weeks post-surgery, end of treatment/withdrawal from the study, and at Visit 1 of the safety follow-up period. (If fecal occult blood + is subject to review, and if fecal occult blood remains + on review, gastroscopy should be performed.), and thereafter as required by the investigator's clinical judgment.

[9] Coagulation function: activated partial thromboplastin time (APTT), prothrombin time (PT), thromboplastin time (TT), fibrinogen (FIB), International Normalized Ratio (INR); performed within 7 days prior to the first dose, 7 days prior to surgical resection, 3-6 weeks post-surgery, end-of-treatment/withdrawal from the study, and at Visit 1 of the safety follow-up period.

[10] Thyroid function: serum thyroid-stimulating hormone (TSH), free triiodothyronine (FT3), free thyroxine (FT4); if FT3 and FT4 are unavailable, T3 and T4 may be substituted; performed within 7 days prior to the first dose, prior to dosing on d1 of the second and third treatment cycles, 7 days prior to surgical resection, 3-6 weeks post-surgery, end of treatment/withdrawal from the study, and at Visit 1 of the safety follow-up period.

[11] 12-lead electrocardiogram: QT, QTc and P-R intervals should be noted. Performed within 7 days prior to the first dose, prior to d1 dosing in the second and third treatment cycles, 7 days prior to surgical resection, 3-6 weeks post-surgery, end of treatment/withdrawal from the study, and at Visit 1 of the safety follow-up period.

[12] Echocardiogram: one examination within 7 days prior to randomization, 7 days prior to surgical resection and at the end-of-treatment visit, plus additional examinations for clinically significant electrocardiographic abnormalities during the study period.

[13] Pregnancy test: For women of childbearing potential, a serum pregnancy test is used. Performed within 7 days prior to first dose, 1 week prior to surgery, 3-6 weeks after surgery, end of treatment/withdrawal from study, and at Visit 1 of the safety follow-up period.

[14] Imaging: including chest CT, abdominal ultrasound; PET-CT testing and cranial magnetic resonance MRI (1.5T scanning + enhancement; cranial CT may be used instead if the patient has a contraindication to magnetic resonance testing) may be performed up to 60 days prior to the first use of the study medication; bone scans will be performed only when clinically indicated, and the bone scans must have been performed within 42 days prior to the first dose of medication.

- ✓ Screening cycle, tumor assessment can be up to 4 weeks prior to first use of study drug, and imaging results obtained prior to signing informed consent can be used for screening period tumor assessment as long as they meet RECIST 1.1 requirements.
- ✓ Full imaging evaluation was performed after the 2nd cycle of dosing and within 7 days prior to surgery for lung cancer.
- ✓ Subjects in whom no imaging progression is observed during safety follow-up and survival follow-up should continue to have imaging evaluations at the same frequency until disease progression or initiation of other antitumor therapy.
- ✓ In addition to disease progression confirmed by imaging, subjects who end treatment for other reasons should also be imaged as often as possible at the frequency specified in the protocol until documented confirmation of disease progression, initiation of new antitumor therapy, or death.

[15] Lung function assessment: Normal or mild to moderate abnormal lung function (VC%>60%, FEV1>1.2L, FEV1%>40%, DLco>40%) to tolerate radical lung cancer surgery; assessed within 14 days prior to the first dose of drug, and once within 7 days prior to lung cancer resection.

[16] Pathologic evaluation: pathologic diagnosis and evaluation within 90 days prior to first dose and after lung cancer resection, respectively.

[17] Adverse events: recorded from the time of signing the informed consent to 90 days after the last dose. Adverse events should be followed until they disappear, resolve to baseline levels or ≤ grade 1, reach steady state, or are reasonably explained (e.g., lost to follow-up, death).

[18] Concomitant medications: record drug combination/concomitant treatments within 14 days prior to the first study medication up to the end of the safety visit, and record drug combination/concomitant treatments only for adverse events related to the use of the study drug after completion of treatment with the study drug or withdrawal from the study.

[19] Safety follow-up: starting after the last study treatment, follow-up visits will be conducted every 30 days (±3 days) until 90 (±7 days) after the last study treatment. The first of these safety visits should be at the study center to complete the examinations and assessments specified in the protocol; subsequent safety follow-ups will be by telephone and will be required only to collect survival information, drug combination/concomitant therapies, and adverse events.

[20] Survival follow-up: at the end of the safety follow-up period, subjects are entered into survival follow-up until the subject dies, is lost to follow-up, informed consent is withdrawn, or the sponsor terminates the study. During this period, follow-up visits were conducted every 3 months during years 1-2, every 6 months during years 3-5, and annually thereafter to collect survival information as well as information on subsequent treatment.

[21] Myocardial enzymes test: creatine kinase isoenzyme (CK-MB), alpha-hydroxybutyrate dehydrogenase, creatine kinase, lactate dehydrogenase, etc., once within 7 days prior to randomization as well as pre-dose, pre-operative, post-operative, and safety follow up for the second and third cycle of the drug; supplemental testing will be performed during the study period in the presence of precordial pain, palpitations, and electrocardiographic abnormalities, as well as at the end of the treatment.

[22] Drug of this study: sintilimab, anilotinib in combination with chemotherapy; see the study dosing section for a detailed description.

List of Abbreviations

Abbreviations and full spelling	Definition
ADLs (activities of daily living)	日常生活活动
AE (Adverse Event)	不良事件
ALT (Alanine Amiotransferase)	丙氨酸氨基转移酶（谷丙转氨酶）
ANC (Absolute Neutrophil Count)	中性粒细胞计数
APTT (Activated Partial Thromboplastin Time)	活化部分凝血活酶时间
AST (Aspartate Aminotransferase)	天冬氨酸氨基转移酶（谷草转氨酶）
BUN (Blood Urea Nitrogen)	尿素氮
Glu (Blood Glucose)	葡萄糖
Cr (Creatinine)	肌酐
CFDA (China Food and Drug Administration)	国家食品药品监督管理总局
CR (Complete Response)	完全缓解
CRF (Case Report Form)	病例报告表
CT (Computed Tomography)	计算机X 射线断层扫描
CTCAE (Common Terminology Criteria for Adverse Events)	不良事件通用术语标准
CK-MB (Creatine Kinase Isoenzyme)	肌酸激酶同工酶
DBIL (Dircet bilirubin)	直接胆红素
ECOG (Eastern Cooperative Oncology Group)	东部肿瘤协作组身体状况评分标准
ECG (Electro Cardio Gram)	心电图
EFS (Event-free survival)	无事件生存期
FT3 (Free-Triiodothyronine)	游离三碘甲状腺原氨酸
FT4 (Free-Thyroxine)	游离甲状腺素
GCP (Good Clinical Practice)	药物临床试验质量管理规范
Hb (Hemoglobin)	血红蛋白
HBV (Hepatitis B Virus)	乙型肝炎病毒
HCV (Hepatitis C Virus)	丙型肝炎病毒
HR (Hazard Ratio)	风险比
INR (International Normalized Ratio)	国际标准化比率
ICF (Informed Consent Form)	知情同意书

IrAE (Immune-related adverse events)	免疫相关性不良事件
MRI (Magnetic Resonance Imaging)	核磁共振
MPR (major pathologic response)	主要病理缓解
MTD (Maximum Tolerated Dose)	最大耐受剂量
NSCLC (Non-small Cell Lung Cancer)	非小细胞肺癌
ORR (Objective Response Rate)	客观缓解率
PD (Progressive Disease)	疾病进展
pCR (Pathological complete response)	病理完全缓解
PI (Principal Investigator)	主要研究者
PLT (Platelet Count)	血小板计数
PR (Partial Response)	部分缓解
PT (Prothrombin Time)	凝血酶原时间
PET/CT (Positron emission tomography computed tomography）	正电子发射计算机断层显像
RBC (Red Blood Cell Count)	红细胞
RECIST (Response Evaluation Criteria in Solid Tumor)	实体瘤疗效评价标准
RFS (Recurrence-free survival)	无复发生存期
SAE (Serious Adverse Event)	严重不良事件
SD (Stable Disease)	疾病稳定
SCLC (Small cell lung cancer)	小细胞肺癌
TBIL (Total Bilirubin)	总胆红素
TSH (Serum Thyroid-stimulating Hormone)	血清促甲状腺激素
TRAE (Treatment-related adverse event)	治疗相关不良事件
ULN (Upper Limit of Normal)	正常值上限
VEGF (vascular endothelial growth factor)	血管内皮生长因子
WBC (White Blood Cell Count)	白细胞

1. Research background and rational for the study

1.1 Research background

1.1.1 Lung cancer and its neoadjuvant therapy

The 2018 Global Cancer Incidence and Mortality Statistics report shows that there are 18.1 million new cancer cases worldwide, of which Asia accounts for nearly half, and of the 9.6 million cancer deaths, Asia accounts for nearly 70%[1]. Lung cancer continues to be the first malignant tumor in terms of incidence (11.6%) and mortality (18.4%), which directly endangers human health. The most common cause of lung cancer is smoking, and lung cancer in never-smokers is more common in women in East Asia and is associated with environmental exposures, including secondhand smoke, pollution, carcinogens, and genetic factors. According to the analysis of lung cancer incidence and deaths in China in 2014, the incidence of lung cancer ranked 1st among malignant tumors in Chinese men and 2nd among malignant tumors in Chinese women, and about 75% of lung cancer patients were found to be in the middle or late stage, with a very low 5-year survival rate[2]. Lung cancer is categorized into non-small cell lung cancer (NSCLC) and small cell lung cancer (SCLC) according to cell type, with NSCLC accounting for about 85% of the total number of lung cancer cases. NSCLC can be divided into three subtypes: adenocarcinoma (40%), squamous cell carcinoma (30%), and large cell carcinoma (15%), which is characterized by slower cell growth and division and later metastasis compared with small cell carcinoma.

Surgical treatment is the main treatment for early stage non-small cell lung cancer, and it is also the only treatment that can make lung cancer cured. It is suitable for stage I-II lung cancer, stage IIIa lung cancer, and partially selective stage IIIb lung cancer whose tumor is confined to one side of the chest cavity. Unfortunately, however, some of the patients with NSCLC in China are in advanced stages when they are found and have missed the best opportunity for surgical treatment. Neoadjuvant therapy, also known as preoperative therapy, mainly refers to the pre-implementation of systemic cytotoxic drug therapy before providing treatment for patients with locally advanced malignant tumors[3]. The standard treatment for patients with stage II disease is surgery followed by adjuvant platinum-based two-agent chemotherapy. Numerous trials and meta-analyses have shown that patients who receive 3-4 cycles of adjuvant

chemotherapy have only a 5% improvement in overall survival. Neoadjuvant therapy has been studied in patients with stage II NSCLC, and preoperative neoadjuvant therapy is usually better tolerated, allowing direct observation of drug efficacy, with no increase in postoperative morbidity or mortality, and an overall survival benefit similar to that of adjuvant therapy[4]. The presence of tumors in mediastinal (N2) lymph nodes is a staging determinant in most stage IIIA patients. However, the time of documented N2 disease (preoperative vs. postoperative) and the extent of N2 disease determine the prognosis and treatment strategy of the patient[5]. Patients with N2 disease have been found to have a worse prognosis when mediastinal lymph node enlargement is observed on CT of the chest and further mediastinal lymph node biopsy is performed, or PET/CT is performed for further evaluation. If these patients undergo direct surgery, the overall 5-year survival rate is 15%. As for neoadjuvant therapy (chemotherapy ± radiotherapy), recent trials have demonstrated an overall 5-year survival rate of 25%. Neoadjuvant therapy followed by surgery for patients with preoperatively diagnosed N2 disease has become one of the standard therapeutic regimens in major clinical guidelines. Since the 1990s, the research syndrome of preoperative neoadjuvant chemotherapy has been increasing year by year, which aims to reduce the scope of the lesion, lower the stage of tumor, increase the rate of complete surgical resection, kill systemic microscopic metastatic foci, avoid recurrence and metastasis, and prolong the survival period. Preoperative neoadjuvant therapy followed by surgery is one of the treatment options for IIIA/N2 and IIIB NSCLC.

1.1.2 Research on molecular markers related to angiogenesis in NSCLC

Tumor angiogenesis is intricately linked to tumorigenesis, progression, and metastasis. As tumors grow, angiogenesis is stimulated to supply nutrients, thereby promoting tumor expansion. Additionally, nascent blood vessels facilitate the dissemination of tumor cells, leading to distal metastases and further disease progression. Anti-angiogenic drugs aim to inhibit this process, thus disrupting the tumor’s nutritional supply, impeding tumor growth, and reducing the likelihood of metastasis[6].

Among various pro-angiogenic factors, vascular endothelial growth factor (VEGF) is a key inducer of tumor angiogenesis, significantly promoting the proliferation and growth of endothelial cells[7]. In lung cancer, VEGF is produced by tumor cells, and

elevated local concentrations of VEGF can upregulate the expression of its receptor, VEGFR, which is a critical mechanism driving angiogenesis in tumor tissues[8]. VEGF facilitates the division and proliferation of vascular endothelial cells and stimulates neovascularization by binding to its primary receptor, VEGFR-2. Research indicates that VEGFR-2 is highly expressed in non-small cell lung cancer (NSCLC) and shows moderate to high expression in lung cancer tissues with lymph node metastases[8,9]. This increased expression of VEGFR-2 is associated with enhanced tumor angiogenesis, accelerated tumor growth, and higher rates of metastasis, contributing to a poorer prognosis for patients.

1.1.3 Neoadjuvant studies of NSCLC and PD-1 inhibitors

Nivolumab shows promising results in neoadjuvant therapy for lung cancer, as evidenced by the CheckMate 159 trial[10]. Nivolumab monotherapy in a neoadjuvant setting has demonstrated a major pathological response (MPR) rate of up to 43%. With a current follow-up of 34.6 months, the median recurrence-free survival (RFS) has not yet been reached. However, the study indicates that patients achieving postoperative MPR have a lower rate of disease recurrence compared to those who do not achieve MPR. In the NADIM trial[11], which investigated neoadjuvant and adjuvant therapy with Nivolumab combined with chemotherapy for stage IIIA NSCLC, there was a significant improvement in pathological complete response (pCR) and MPR. The objective response rate (ORR) was 78.5%, all patients underwent R0 resection, and 93% experienced postoperative pathological downstaging. These findings suggest that neoadjuvant immunotherapy holds great potential in the treatment of lung cancer. However, further studies are necessary to fully explore and validate the efficacy and safety of neoadjuvant immunotherapy in this context.

1.2 Rational for the study

With the widespread use of preoperative neoadjuvant therapy in the clinic, research on the application of antiangiogenic drugs in preoperative neoadjuvant therapy for locally advanced NSCLC continues to receive attention[12]. Anlotinib hydrochloride is a multi-targeted receptor tyrosine kinase inhibitor with significant inhibitory activity against several kinases involved in angiogenesis and tumor proliferation. Specifically, it targets VEGFR1/2/3, FGFR1/2/3, and other tumor-related kinases such as PDGFRα/β, c-Kit, and Ret kinases, offering a broad spectrum of inhibition that extends

to angiogenic kinases like Met and FGFR1/2/3[7]. Additionally, Anlotinib exhibits potent inhibitory effects on several kinases currently under study, including Aurora-B, c-FMS, and DDR1. It is also effective against a variety of kinase mutants. By inhibiting tumor angiogenesis and promoting the normalization of blood vessels, Anlotinib disrupts the nutritional supply to tumors, thereby inhibiting tumor growth and reducing the likelihood of metastasis. It has been approved for the treatment of a variety of tumors in China, including locally advanced or metastatic NSCLC and SCLC with progression or recurrence after systemic chemotherapy, alveolar soft tissue sarcoma, clear cell sarcoma and advanced soft tissue sarcoma with progression or recurrence after chemotherapy. Its efficacy and safety have been observed in a number of clinical studies of first- and third-line treatment of lung cancer[8]. In 2015, ASCO published a study of bevacizumab with chemotherapy in preoperative neoadjuvant chemotherapy for patients with lung adenocarcinoma, enrolling 42 patients with locally advanced stage IIIA (N2) and IIIB lung adenocarcinomas, which showed that the regimen was effective in increasing the rate of surgical resection and had a good safety profile[13]. Neoadjuvant therapy for resectable NSCLC has become one of the recognized effective means. In neoadjuvant therapy, the main therapeutic tools include chemotherapy, TKI, radiotherapy, etc. The above studies have shown that the postoperative pCR is about 0-17%, and that the postoperative pCR is closely related to postoperative patient OS[5]. Recently, immunotherapy has made impressive achievements in neoadjuvant therapy for lung cancer, and CheckMate 159 trial suggests that Nivolumab significantly improves PCR and MPR rates in preoperative neoadjuvant therapy, has better PFS, and is expected to improve OS. The existing neoadjuvant treatments of atalizumab in combination with chemotherapy and Nivolumab in combination with chemotherapy have achieved impressive results and high PCR. Therefore, in the neoadjuvant treatment of NSCLC, we attempted to carry out this neoadjuvant treatment of "quadruple combination (sintilimab, anlotinib combined with platinum-containing two-agent chemotherapy)", and observed the efficacy and safety of this clinical program.

2. Research objectives

To evaluate the efficacy and safety of neoadjuvant therapy for IIA-IIIB NSCLC by sintilimab and anlotinib in combination with chemotherapy.

3. Study Design

3.1 Description of research design

This is a multicenter, prospective, open, single-arm clinical study to preliminarily explore the efficacy and safety of neoadjuvant sintilimab and anlotinib in combination with chemotherapy for IIA-IIIB NSCLC.

3.2 Primary endpoints

Efficacy: Observe the pathological complete response (PCR) rate of resected tumor tissues in postoperative patients

Safety: According to NCI-CTCAE version 5.0, closely observe and record the incidence, severity, association, risk factors, measures taken and their regression of all adverse events (AEs); special attention should be paid to AEs of special interest (AESIs), which mainly include immune-associated pneumonia, immune-associated cardiomyopathy, immune-associated endocrine system disorders, hypertension, hemorrhage and proteinuria.

3.3 Secondary endpoints

Observe the postoperative major pathologic response (MPR) rate, event-free survival (EFS) and overall survival (OS).

3.4 Sample size

The study was planned to be conducted in the Department of Thoracic Surgery of Tangdu Hospital of Air Force Medical University and the Third Affiliated Hospital of Chongqing Medical University, and a total of 45 patients were recruited.

3.5 Inclusion and exclusion criteria

3.5.1 Inclusion criteria

Subjects must meet the following inclusion criteria:

1) Age ≥ 18 and ≤ 75 years with a ECOG PS score of 0-1;

2) Subjects who can adhere to the study protocol;

3) At least one measurable lesion according to RECIST 1.1 criteria;

4) Stage IIA, IIB, IIIA, and IIIB NSCLC with histologically or cytologically

confirmed resectable NSCLC according to International Association for the Study of Lung Cancer and the 8th edition lung cancer TNM staging of American Joint Committee on Cancer Staging;

5) Participants must have a tumor tissue sample available for PD-L1 immunohistochemistry (IHC) and must submit a formalin-fixed, paraffin-embedded (FFPE) tissue block or unstained tumor tissue section and corresponding pathology report prior to enrollment. Tissue must be biopsy specimens from hollow-needle aspiration biopsies, excisions, or incisions. Fine-needle aspiration biopsies obtained by EBUS are insufficient for biomarker review and as a basis for randomization.

6) Tumor tissue samples must be fresh or obtained within 3 months prior to enrollment.

7) For all suspicious mediastinal lymph nodes evaluated by PET-CT or contrast CT findings, a pathological diagnosis was not necessarily required.

8) Good hematopoietic function, defined as absolute neutrophil count ≥ 1.5×109/L (not receiving granulocyte colony-stimulating factor supportive therapy), platelet count ≥ 100×109/L, hemoglobin ≥ 90 g/L; (no transfusion or no erythropoietin dependence within 7 days);

9) Good liver function, defined as serum total bilirubin ≤ 1.5 times the upper limit of normal (ULN); glutamic oxaloacetic transaminase (AST) and glutamic pyruvic transaminase (ALT) levels ≤ 2.5 times ULN in patients without hepatic metastases; and AST and ALT levels ≤ 5 times ULN in patients with documented hepatic metastases;

10) Good renal function, defined as serum creatinine ≤1.5 times ULN or calculated creatinine clearance ≥60 ml/min (Cockcroft-Gault formula); urine protein less than 2+ on routine urinalysis, or 24-h urine protein <1 g;

11) Good coagulation function, defined as an international normalized ratio (INR) or prothrombin time (PT) ≤ 1.5 times ULN;

12) Total lung function (e.g., FVC, FEV1, TLC, FRC, and DLco) capable of tolerating the proposed lung resection, as assessed by the surgeon;

13) For female subjects of childbearing potential, a negative urine or serum pregnancy test should be performed within 3 days prior to receiving the first dose (Cycle 1, Day 1);

14) If there is a risk of conception, male and female patients are required to use highly effective contraception and continue until at least 180 days after discontinuation of treatment.

3.5.2 Exclusion criteria

1) Presence of locally advanced unresectable or metastatic disease;

2) Participants with known ROS1 fusion-positive or ALK translocations;

3) ≥ grade 2 peripheral neuropathy;

4) Active autoimmune diseases (known or suspected). Type I diabetes, hypothyroidism requiring only hormone replacement therapy, skin disorders that do not require systemic therapy (e.g., vitiligo, psoriasis, or alopecia areata), or disorders that are not expected to recur in the absence of external stimuli are eligible for enrollment;

5) Those requiring systemic treatment with glucocorticoids (>10 mg prednisone equivalent dose per day) or other immunosuppressive drugs within 14 days prior to randomization. Inhaled or topical steroids are permitted in the absence of active autoimmune disease;

6) Active hepatitis B/hepatitis C infection and known HIV infection;

7) Patients with prior chemotherapy or any other anti-tumor therapy;

8) Patients having received the following therapies in the past: anti-PD-1, anti-PD-L1, anti-PD-L2, or anti-CTLA-4 antibodies or any other antibody targeting T-cell co-regulatory pathways;

9) Patients who have been treated with anti-angiogenic drugs, such as anlotinib;

10) Patients with the presence of uncontrollable hypertension;

11) Presence of clinically uncontrollable pleural effusion or abdominal effusion;

12) Presence of clinically active diverticulitis, abdominal abscess, gastrointestinal obstruction;

13) Have received a solid organ or blood system transplant;

14) Diagnosis of other malignancies within 5 years prior to the first dose, excluding carcinoma that have been apparently cured, such as basal or squamous cell skin cancer, superficial bladder cancer, or carcinoma in situ of the prostate, cervix, or

breast;

15) Have active infection requiring systemic therapy;

16) Vaccination within 30 days prior to the first dose (Cycle 1, Day 1);

17) Patients with hemoptysis (>50 mL/day);

18) Patients whose tumors have invaded important blood vessels or who are at risk of hemorrhage during subsequent studies.

3.5.3 drop-out/eliminate criteria

1) Failure to administer the medication in accordance with the dosage, method and course of treatment specified in this research program.

2) Note: Cumulative discontinuation of more than two weeks in a dosing cycle is recorded as drop-out. Failure to use the medication in accordance with the provisions of the protocol resulting in the inability to conduct efficacy and/or safety evaluations;

3) Serious violation of the protocol: concurrent application of chemotherapeutic agents and/or treatment with modern Chinese medicinal preparations with anti-lung cancer indications while participating in this study.

3.5.4 Termination criteria

1) Disease progression;

2) Grade 3/4 adverse events failure to complete treatment after dose adjustment according to the trial protocol;

3) Death;

4) Occurrence of unanticipated and unacceptable adverse drug reactions;

5) Delayed dosing for >4 weeks. Serious violation of the trial protocol (including serious violation of study enrollment conditions).

3.5.5 Withdrawal Criteria

Patients can withdraw from the study at any time, without giving a reason.

Patients must withdraw from the study if any of the following events occur:

1) Patient voluntarily withdraws from the study.

2) Subject or his/her legal representative requests to withdraw from the study;

3) There are medical or ethical reasons affecting the continuation of the study;

4) The investigator determines that withdrawal from the study is necessary in the best interest of the subject;

5) The subject loses to follow-up;

6) Subject pregnancy;

7) Serious violation of the trial protocol (including serious violation of study enrollment criteria).

3.6 Treatment management

3.6.1 Treatment plan

Sintilimab (200mg fixed dose) iv, d1, q3w + anlotinib 10mg, po, qd1-14, q3w + platinum-doublet chemotherapy, evaluated after 3 cycles of combination therapy and discontinued for 3 weeks (21 days) followed by surgery, which was performed within 4-6 weeks (22-42 days) of the last dose. The time between each study dosing treatment was called a cycle.

A 3-week dosing and observation cycle was established for the trial. Patients who achieved effective disease control, including complete response (CR), partial response (PR), or stable disease (SD), continued treatment until surgery as per the trial protocol (Tumor Assessment Form, according to RECIST 1.1). For patients who experienced intolerable toxicity, disease progression, or requested to discontinue the drug, treatment was terminated. In cases of disease progression confirmed by imaging, the principal investigator (PI) should promptly review and understand the situation. If certain conditions are met, the patient may still be eligible for surgery with the PI’s consent: the patient must be able to tolerate the surgery in terms of symptoms or quality of life, be fully informed and willing, and the surgery should be assessed by the treating physician (investigator) as potentially providing a survival benefit. If surgery is not deemed suitable, the patient may instead undergo standard radiotherapy treatment with the PI’s consent.

NSCLC			
**Drugs in the research**	**Mode of administration**	**Frequency**	**Duration of treatment***
Sintilimab (200mg fixed dose)	Intravenous, over 30 minutes	Day 1 (one cycle every 3 weeks)	9 weeks
Anlotinib 10mg	Oral	Daily (2 weeks on, 1 week off)	9 weeks
Platinum-doublet chemotherapy (guideline recommended drug)	Intravenous	Day 1 (one cycle every 3 weeks)	9 weeks

*1 cycle = 3 weeks, treatment duration = 3 cycles

The medication record (Sintilimab and anlotinib) is confirmed through the following table.

A Prospective Single-arm, Open-label Study of Sintilimab and Anlotinib Combined with Chemotherapy in Neoadjuvant Treatment of Resectable NSCLC
Research Center Name/No.: The Second Affiliated Hospital of Air Force Medical University (Tangdu Hospital)/01
Principal Investigator: Xiaolong Yan

Sintilimab Configured Infusion Destruction Record Sheet

(1) Basic Information

Subject No.: ________ Initials: ________ Cycle ______

Planned dose administered: _______ mg Actual dose administered: _______ mg

Size 100 mg/vial ___ vial used: Drug No.: ________

(2) Drug dispensing records

Dilution date: ___ _______

Dilution start time: ___ ___ Dilution end time: ___ ___

Sintilimab: ___ ml Dilution to 0.9% sodium chloride injection: ___ ml;

Signature of operator: ________

(3) Drug infusion records

Infusion time: ___ ___ to ___ ___ Signature of operator: ________

(4) Drug destruction

If you need to destroy at the center, please fill in the destruction information, otherwise, please check: □ Not Applicable

Destruction time: ___ ___ ___ Quantity: ___ vials (including empty vials) Signature of destroyer: ________

Record card of Anlotinib

Subject No.:				Patient :_________					
1st treatment cycle	Week 1	Date							
		Whether or not to take							
	Week 2	Date							
		Whether or not to take							
	Week 3	Date							
		Whether or not to take							
2nd treatment cycle	Week 1	Date							
		Whether or not to take							
	Week 2	Date							
		Whether or not to take							
	Week 3	Date							
		Whether or not to take							
3rd treatment cycle	Week 1	Date							
		Whether or not to take							
	Week 2	Date							
		Whether or not to take							
	Week 3	Date							
		Whether or not to take							

Precautions: This table is a record card for the dosing of Anlotinib, which is taken once daily, 1 capsule/dose, before breakfast on an empty stomach. Use for two weeks and stop for one week. If the dose is missed, it cannot be made up within 12 hours of the next dose.
Registration requirements: Please fill in the date according to the facts, "Whether or not to take" please tick or "×". The gray part means that the drug will not be taken for one week.

3.6.2 Dose adjustment and discontinuation of administration

Anlotinib was discontinued if patients developed haemoptysis. For other grade ≥3 treatment-related adverse events (TRAEs), anlotinib was reduced to 8 mg, and the dose of chemotherapy was reduced by 25.0% of the last dose. Dose modification of sintilimab was prohibited. Patients experiencing intolerable adverse events that led to a delay or discontinuation of one drug continued treatment with the remaining study drug. Treatment continued until intolerable toxicity occurred or consent was withdrawn. Those who developed progressive disease were permitted to undergo surgery or receive SOC-CRT at the investigator's discretion.

3.6.3 Drug management

Sintilimab injection should be stored in the original package with the vial under refrigeration at 2~8°C, protected from light, avoiding refrigeration and shock, with an expiration date of 24 months. Anlotinib hydrochloride capsule should be kept under light shielding, airtight, and below 25°C, with an expiration date of 18 months.

3.6.4 Treatment prescription

Treatment prescription was prescribed by the patient's physician, and patients suitable for this study were selected for voluntary enrollment based on the enrollment criteria.

3.7 Duration of research

The study is expected to last 5 years, including a 12-month enrollment period. After discontinuation of treatment, each patient will be followed-up until death or withdrawal from the study.

- Planned date for 1st patient enrollment/trial start: June 10, 2021
- Planned date for the last patient enrollment: June 10, 2023
- Planned date for the last patient to be disenrolled/end of trial: October 10, 2023 (4 months after enrollment of last eligible patient)
- Expected report date: December 30, 2023

4. Research procedures and data collection

4.1 Data collection program

The duration of safety data collection spans from the time the patient signs the Informed Consent Form (ICF) until 30 days after the final dose. All adverse events must be documented in the Case Report Form (CRF) from the moment the ICF is signed until the study concludes. The incidence and severity of adverse events will be observed and assessed according to the NCI-CTCAE 5.0 criteria. Each patient will undergo scheduled visits, and specific data will be recorded at various time points during these visits.

4.2 Screening phase

The screening period for this study will be completed within 28 days. During this time, additional study visits may be included due to variations in study procedures. At the screening visit, patients will be asked to read and sign the informed consent form before undergoing any procedures related to the study that are not part of standard care. As research subjects, patients have the right to be fully informed about the study and may request the doctors to explain or clarify any part of the informed consent form that they do not understand. The study staff will perform the following tests and procedures to confirm the patient’s eligibility to participate in this study:

1) Review patients’ medical history;

2) Review of medications the patients are currently taking and have taken in the past, including herbal medications;

3) Have a physical exam that includes testing height, weight, body surface area (BSA) and vital signs (temperature, blood pressure, respiration, and heart rate), pulse,

oxygen saturation by oximeter, and pulmonary function tests. An electrocardiogram (ECG) will be performed prior to the start of study treatment;

4) The patients will be asked about symptoms related to the disease (performance status) that they are suffering from;

5) Imaging:

Positron emission tomography/computed tomography (PET/CT) scans will be conducted from the base of the skull to the upper thighs. During this procedure, patients will be injected with a radioactive tracer that is absorbed by cancer cells, allowing for their detection by the PET scanner. Additionally, computed tomography (CT) will be used to image the internal structures of the body. CT scans and magnetic resonance imaging (MRI) will be performed on areas of known disease.

6) Sample collections:

A blood sample (approximately 20 mL) will be collected for laboratory testing. This will include assessments of blood chemistry, kidney and liver function, as well as counts of red blood cells, white blood cells, and platelets. Thyroid function will also be tested, and patients will be screened for Hepatitis B Virus (HBV) and Hepatitis C Virus (HCV) infections. Patients wishing to participate in the study must be free of known HIV infection and must test negative for HBV and HCV or have an HBV viral load of ≤ 2000 IU. For female subjects of childbearing potential, a urine or blood sample will be required for a pregnancy test. Additionally, a sample of tumor tissue will be tested for PD-L1 expression. If no tumor tissue is available, the patient will be asked to undergo a tumor tissue biopsy for a more precise assessment. At least 10 tissue sections will be needed.

7) Sample testing

Doctors may decide to test tumor tissue samples for ROS1 or ALK gene mutations. PD-L1 Testing: Part of this study involves evaluating whether patients with a specific biomarker are likely to respond to the study treatment. During the screening process, biomarker testing will be conducted to identify certain genes, which will enable the study doctor and clinical team to determine the patient's eligibility for participation. This biomarker testing may also provide valuable information to help guide research decisions based on the genetic profile of the participant's disease.

4.3 Preoperative treatment period

Sintilimab (200mg fixed dose) iv, d1, q3w + anlotinib 10mg, po, qd1-14, q3w + platinum-doublet chemotherapy, evaluated after 3 cycles of combination therapy and discontinued for 3 weeks (21 days) followed by surgery, which was performed within 4-6 weeks (22-42 days) of the last dose. The time between each study dosing treatment was called a cycle.

4.4 Radical surgery

Within 4-6 weeks (22-42 days) of the last dose, the radical surgery was performed.

4.5 Follow-up period

After completing the pre-surgical treatment, patients will enter a follow-up period. During this time, doctors will continue to evaluate the patients' health. It is crucial to monitor their health status, including recovery progress, potential disease progression, and any serious adverse events. Patients may undergo surgery to remove tumor tissues during this period. Some of the tumor samples will be analyzed to assess how much of the tumor remains and to test for the same or similar biomarkers that were evaluated during the screening phase.

Initial follow-up *(1st and 2nd follow-up)*

The first follow-up visit will take place in the hospital approximately one month after the completion of pre-surgical treatment, and it will occur before or on the day of the lumpectomy. Surgery is scheduled to be performed within 4-6 weeks after finishing pre-surgical treatment. Prior to surgery, a contrast-enhanced PET/CT or CT scan will be conducted, and the results of pulmonary function tests will be reassessed. A second follow-up visit will occur approximately two months after the first follow-up visit and after the patient has undergone surgery. Procedures performed and samples collected during the study drug administration may be repeated one or more times during these two follow-up visits. Following the initial follow-up visit and surgery, patients will receive adjuvant therapy with sintilimab for up to 13 cycles as part of their standard care.

4.6 Additional follow-up

After surgery, patients will have additional follow-up visits approximately every three months, with the possibility of more frequent check-ins. These follow-up visits

can be conducted over the phone or in the hospital. Patients will receive evaluations every three months for a total of two years, followed by visits every six months thereafter. As part of the follow-up, patients will undergo a CT or MRI scan of the lesion site or areas of potential spread every three months for the first two years. This will continue every six months for the next three years and then annually for a total of five years, until tumor recurrence or death occurs.

4.7 Survival visit

After surgery, follow-up visits will be conducted every three months for the first two years, followed by visits every six months for an additional three years. Follow-up care includes, but is not limited to, performing CT or MRI scans of the lesion site or areas of potential spread until tumor recurrence or death occurs. During follow-up, the following parameters will be closely monitored and recorded: time to disease progression, time of death (with options for telephone follow-up and record-keeping), and any other therapeutic agents or treatments administered.

4.8 Adverse event follow-up

After the last dose, follow-up will continue to monitor for any new adverse events (AEs). For AEs that do not resolve after discontinuation of the drug, monitoring will continue until the adverse event is resolved, returns to baseline levels, or is deemed clinically insignificant.

4.9 Handling steps for patient withdrawal from follow-up program

Patients have the right to withdraw informed consent at any time and for any reason. If a patient agrees to participate in survival follow-up, they will not be considered to have withdrawn from the study. However, if they choose not to participate in survival follow-up, they will be considered withdrawn. Therefore, investigators should make every effort to regularly contact patients to assess their health status, including at least their survival status.

5. Statistical methods

5.1 Analysis variables

5.1.1 Main indicators

1) Pathologic complete response (pCR) rate: The pCR rate is defined as the proportion of subjects with no residual invasive cancer on hematoxylin and eosin-stained slides of resected lung specimens and lymph nodes after completion of neoadjuvant therapy. For pathological assessments, primary lung tumors and lymph node specimens were staged using the AJCC criteria (8th edition). Residual viable tumor percentage in primary tumors was determined from routine hematoxylin and eosin-stained specimens. Tumors with no residual tumor cells (ypT0N0M0) were classified as having pCR. Pathological downstaging was defined as a reduction in ypTNM stage post-treatment, with no new lesions or progression of existing lesions.

2) Safety

- Incidence of treatment-related adverse events (TRAE);
- Incidence of AEs that resulted in treatment discontinuation and/or early study withdrawal;
- Incidence of SAEs and drug-related SAEs;
- Incidence of deaths;
- Discontinuation of medication for adverse event reasons;
- Changes in vital signs before and after treatment (heart rate, blood pressure, temperature, respiration);
- Changes in whole body physical examination before and after treatment;
- Abnormalities in clinical laboratory tests.

5.1.2 Other indicators

- Major pathologic response (MPR) rate, defined as the proportion of subjects with ≤10% surviving tumor cells in the resected primary tumor and all resected lymph nodes. For pathological assessments, primary lung tumors and lymph node specimens were staged using the AJCC criteria (8th edition). Residual viable tumor percentage in primary tumors was determined from routine hematoxylin and eosin-stained specimens. Tumors with ≤10% viable cells were classified as having MPR. Pathological downstaging was defined as a reduction in ypTNM stage post-treatment, with no new lesions or progression of existing lesions.
- Event-free survival (EFS), defined as the interval from enrolment to the

earliest occurrence of local progression resulting in inoperability; unresectable tumour, disease progression or recurrence according to RECIST version 1.1 as assessed by the investigator; or death from any cause. Progression-free survival was treated as censored on the date of the last validated tumor assessment for subjects in whom no tumor progression or death was observed.

- Overall survival (OS), defined as the time from study entry (i.e., after signing ICF) to death from any cause. Subjects who were alive at the time of last contact had their overall survival censored at the date of last contact.

- Patients underwent radiological evaluations by investigators according to RECIST version 1.1 at baseline, after two cycles of neoadjuvant therapy, prior to surgical resection, and every 4 cycles during adjuvant therapy until disease progression, intolerable toxicity, death, or withdrawal of consent. Complete and partial responses were confirmed radiologically at least 4 weeks later, while stable disease was confirmed at least 8 weeks after the initial assessment. The ORR was defined as the proportion of patients achieving either a complete or partial response. Patients were followed up by visits or phone calls every 12 weeks to determine survival status.

Tumor Assessment Form（RECIST 1.1 ）

**Center No.:**	**Center Name:**						**Subject initials:**			**Subject No::**		
**Principal Investigator:**				**Screening period**			**Upon completion of two cycles of neoadjuvant**			**Preoperative evaluation (week from C1D1)**		
Examination methods 1. plain CT 2. enhanced CT or plain + enhanced CT 3. plain MRI 4. enhanced MRI or plain + enhanced MRI 5. bone scan 6. PET 7. PET-CT 8. ultrasound 99. others, please specify												
**Target lesion**												
Lesion No.	Lesion location: Anatomical site (left lung,	Specific part	Inspection Method No.	Inspection date	Is the layer	Longest diameter (non-lymph node lesions) or	Inspection date	Is the layer	Longest diameter (non-lymph node lesions) or	Inspection date	Is the layer	Longest diameter (non-lymph node lesions) or shortest diameter
01						mm			mm			mm
02						mm			mm			mm
03						mm			mm			mm
04						mm			mm			mm
05						mm			mm			mm
**Sum of the longest/shortest diameters of all target lesions**						**mm**			**mm**			**mm**
**Assessment of target lesion efficacy: comparison with baseline or minimum**				NA			□ Increase □ Decrease ____ %			□ Increase □ Decrease ____ %		
**Non-target lesion status:**				£Yes £No			£Yes £No			£Yes £No		
Lesion No.	Lesion location: Anatomical site (left lung,	Specific part	Inspection Method No.	Inspection date	Is the layer	Non-target lesion status	Inspection date	Is the layer	Non-target lesion status	Inspection date	Is the layer	Non-target lesion status
Non-target lesion status: Please check options: □ A present □ B absent □ C significantly progressed □ D unable to assess □ E not done												
01						□ A □B □C □D □E			□ A □B □C □D □E			□ A □B □C □D □E
02						□ A □B □C □D □E			□ A □B □C □D □E			□ A □B □C □D □E
03						□ A □B □C □D □E			□ A □B □C □D □E			□ A □B □C □D □E
04						□ A □B □C □D □E			□ A □B □C □D □E			□ A □B □C □D □E
05						□ A □B □C □D □E			□ A □B □C □D □E			□ A □B □C □D □E
**Non-target lesion efficacy evaluation: CR Non-CR/Non-PD PD NE NA**				NA			□CR □Non-CR/Non-PD □PD			□CR □Non-CR/Non-PD □PD		
**Any new lesions since last imaging test**				NA			£Yes £No □NE			£Yes £No □NE		
Lesion No.	Lesion location: Anatomical site	Specific part	Inspection Method No.	NA			Inspection date	Is the layer	Type of pathology (if done)	Inspection date	Is the layer	Type of pathology (if done)
				NA								
				NA								
				NA								
**Any splitting or fusion of lesions since last imaging test**				NA			£Yes £No			£Yes £No		
Description of lesion splitting or fusion (please record the status of splitting or fusion				NA			Description:			Description:		
**Overall efficacy assessment: CR; PR; SD; PD; NE**				NA			□CR □PR □SD □PD □NE			□CR □PR □SD □PD □NE		
**Signature of investigator/authorized imaging physician and date**												

5.2 Statistical methods

5.2.1 General principles

A statistical analysis plan was developed by the biostatistician and the principal

investigator based on the study protocol, and the formative documents were refined before data locking.

The statistical analysis software was SPSS software version 19.0, and the confidence level for all confidence intervals was taken as 95%.

For continuous variables, the number of non-missing subjects, mean, standard deviation, median, minimum and maximum values will be listed. For categorical variables, they will be listed in the form of frequency tables (frequencies and percentages).

5.2.2 Principal analysis

The primary endpoints were the statistical rate of complete pathological remission, and the incidence, severity, association, risk factors, measures taken and their regression for all AEs.

5.3 Determination of sample size

Number of subjects: n=45

The sample size calculation used Simon's two-stage maximum value design. Based on the Checkmate-816 trial[14], the minimum pCR rate was set at 25%, and the expected pCR rate was 45%. Assuming a type I error rate (α) of 0.05 and a type II error rate (β) of 80%, a sample size of 41 patients was needed. Seventeen patients were enrolled in stage 1. If more than 5 patients achieved pCR, the trial proceeded to stage 2; otherwise, the trial was terminated.

6. Adverse event reporting

6.1 Adverse event observation

An adverse event (AE) is any unfavorable medical occurrence that happens after a subject in a clinical trial has signed an informed consent form, regardless of whether it is causally related to the treatment. AEs can encompass a wide range of unfavorable symptoms, signs, laboratory test abnormalities, or diseases. Specifically, AEs include at least the following:

1) A pre-existing medical condition or disease is recorded as an AE only if it worsens after the initiation of study treatment.

2) Any newly occurring AE: This includes any new adverse medical condition (such as symptoms, signs, or newly diagnosed diseases).

3) Abnormal clinically significant laboratory test results that are not attributable to concomitant diseases.

Adverse events that occur during both the pre-treatment and post-treatment phases are also considered AEs according to regulations. Therefore, safety monitoring for AEs or serious adverse events (SAEs) should be reported from the time the subject enters the trial (by signing the informed consent form) until the end-of-trial visit.

6.2 AE rating

AEs are categorized as grade 1-5 according to NCI Classification Criteria for Common Acute and Subacute Toxicities (NCI-CTCAE version 5.0). If the AE is not listed in the NCI toxicity classification criteria, it can be judged according to the following criteria:

1) Grade 1 (mild): no clinical symptoms or mild clinical symptoms; abnormal clinical or laboratory tests only; no treatment required;

2) Grade 2 (moderate): requires minor, localized or non-invasive treatments; age-appropriate limitations in Activities of Daily Living (ADLs), which are defined as cooking, shopping, talking on the phone, counting money, etc;

3) Grade 3 (severe): a serious adverse event (SAE) may include a condition that is severe or has medically significant symptoms but is not currently life-threatening. It can result in hospitalization or prolonged hospitalization, lead to disability, and restrict the individual's ability to perform activities of daily living (ADLs). Self-care ADLs encompass tasks such as bathing, dressing, undressing, eating, using the bathroom, and taking medication, as long as the individual is not bedridden;

4) Grade 4 (severe): Life-threatening and requires urgent treatment;

5) Grade 5 (death): Lethal.

6.3 AE record

Detailed records of the name, severity, time of occurrence, duration, management measures, and resolution of various adverse events (AEs) during the study period will be meticulously documented in the case report form (CRF). Related examinations will

be conducted at least once a week and followed up until normalization or the end of the study. Any adverse events occurring within 30 days of the last dose should be reported and recorded.

6.4 Determination of the relationship between AEs and the test drug

Possible associations between AEs and the test drug were evaluated according to a five-tier classification, including: definitely related, very probably related, probably related, probably not related, and not related.

6.5 SAE

(1) Definition of serious adverse event

A serious adverse event (SAE) refers to medical occurrences during clinical studies that necessitate hospitalization or prolonged hospitalization, result in disability, impact the ability to work, endanger life, or lead to death or congenital malformations. SAEs primarily include the following unintended medical events:

Events that result in death;

Life-threatening events (the term "life-threatening" means that there is an immediate risk of death to the subject at the time of the event/reaction; it does not mean that death may occur only if the event/reaction deteriorates further);

Events that require hospitalization or prolonged hospitalization;

Events that can result in permanent or severe disability/malfunction;

Congenital anomalies or birth defects;

Other important medical events.

(2) Pregnancy

Pregnancy occurring during a clinical study should be reported as SAE.

(3) Disease progression

Disease progression (including signs and symptoms of progression) should not be reported as an SAE. But the death due to disease progression occurring within the trial or safety reporting period should be reported as an SAE. Hospitalization for signs and symptoms of disease progression should not be reported as an SAE. If the final

outcome of the cancer is death within the trial or safety reporting period, then the event leading to death is reported as an SAE.

(4) Perform other anti-tumor therapy

If the subject is initiated on other antitumor therapy, the reporting period for non-death AEs ends with the initiation of the new antitumor therapy. If the death occurs within the SAE reporting period after the end of study treatment, it must be reported regardless of whether the patient receives other treatment.

(5) Hospitalization for treatment

AEs that result in hospitalization or prolonged hospitalization in clinical studies should be considered SAEs. Any initial admission to a healthcare facility (even if shorter than 24 hours) meets this criterion.

Hospitalization does not include the following conditions:

Rehabilitation facilities or convalescent hospital;

Routine emergency room admissions;

Same-day surgery (e.g., outpatient/same-day/ambulatory) Inpatient hospitalization unrelated to worsening of AE or prolonged hospitalization is not an SAE;

Admission for a pre-existing condition with no new AE occurring and no aggravation of the pre-existing condition (e.g., to screen for abnormal laboratory tests that have persisted to date prior to the test);

Hospitalization for administrative or medicare-insured reasons (e.g., routine annual physical examination);

Hospitalization specified by the trial protocol during the clinical study (e.g., operation as required by the trial protocol);

Elective hospitalizations not related to worsening of AE (e.g., elective cosmetic surgery);

Scheduled treatments or surgical procedures should be documented throughout the trial protocol and/or in the individual subject's baseline information;

Hospitalization solely for blood product use.

Diagnostic or therapeutic invasive (e.g., surgery), non-invasive procedures should not be reported as AEs. However, if the disease condition that led to the procedure meets the definition of an AE, it should be reported.

(6) SAE reporting procedures

SAEs should be reported from the time the subject signs the informed consent form until the 30th (including) calendar day after the last dose of study drug. If a serious adverse event occurs during the trial, it must be reported to the clinical supervisor and the principal investigator within 24 hours. At the same time, Serious Adverse Event (SAE) Report Form for Clinical Research must be filled out, signed and dated, and immediately reported to the sponsoring organization, the ethics committee of the research unit, CFDA, and the Food and Drug Administration of the region where the investigator is located (either provincial or municipal), in the form of a facsimile transmission.

Information on all SAEs must be recorded on the SAE form. Serious adverse events occurring during the continuation period up to 30 days after the last dose must be reported. SAEs occurring after 30 days of the last dose are generally not reported unless they are suspected to be related to the study drug.

For SAEs, detailed observation and documentation of symptoms, severity, time of occurrence, time to management, measures taken, time and mode of follow-up, and regression should be documented. If the investigator believes that a serious adverse event is not related to the trial drug, but is potentially related to a study condition (e.g., discontinuation of the original treatment, or comorbidities during the course of the trial), this relationship should be detailed in the narrative section of the SAE on the medical record report form. If there is a change in the intensity of an ongoing SAE or its relationship to the subject drug, a serious adverse event follow-up report should be sent to the sponsor immediately. All SAEs should be followed until recovery or stabilization.

6.6 irAE

Immune-related adverse events (irAE) are specific events (including: pneumonia, diarrhea/colitis, hepatitis, nephritis/renal dysfunction, rash, and endocrinopathies) for which subjects received immunosuppressive medications for treatment. Endocrine events (hypothyroidism/thyroiditis, hyperthyroidism, pituitary gland inflammation,

diabetes mellitus, adrenal insufficiency) are usually exceptions because these endocrine events are generally not related to treatment and can usually be managed without immunosuppressive interventions. Preferred terms used to support the warnings and precautions included in irAE analysis are shown in the table below:

**irAE category**	**Terms included under irAE category (MedDRA codes)**
Pneumonitis	Pneumonitis, interstitial pneumonitis
Diarrhea/Colitis	Diarrhea, colitis, enterocolitis
Hepatitis	Hepatotoxicity, hepatitis, acute hepatitis, autoimmune hepatitis, elevated AST, elevated ALT, elevated bilirubin, elevated ALP
Adrenal Insufficiency	Adrenal insufficiency
Hypothyroidism/Thyroiditis	Acute thyroiditis (collapse with the frequency of thyroiditis), autoimmune thyroiditis (collapse with the frequency of thyroiditis)
Hyperthyroidism	Hyperthyroidism
Pituitary gland inflammation	Pituitary gland inflammation
Diabetes	Diabetes, diabetic ketoacidosis
Nephritis and renal dysfunction	Nephritis, acute nephritis, tubulointerstitial nephritis, acute renal failure, renal failure, elevated creatinine
Rash	Rash, maculopapule

6.7 Postoperative complication

Postoperative complications were evaluated using the Clavien–Dindo Classification of Surgical Complications.

7. Quality control and quality assurance

Personnel participating in this study, including clinicians and supervisors, will be rigorously trained to ensure that the trial is performed in accordance with the clinical study protocol, to protect the safety and rights of the subjects, to follow standard operating procedures, and to correctly and completely record and report study data.

8. Ethical, regulatory and administrative principles

8.1 Ethical principles

This study will be conducted in accordance with the principles established by the 18th Joint World Medical Association Congress (Helsinki, 1964) and all subsequent revisions.

8.2 Laws and regulations

This study will be conducted in compliance with relevant national laws and regulations and the current GCP guidelines of the CFDA.

8.3 Data protection

Personal data of patients and personal data of investigators that may be included in the Company's database shall be handled in accordance with relevant national laws and regulations. When archiving or processing personal data related to investigators and/or patients, care must be taken to protect the privacy of individuals and appropriate measures must be taken to protect and prevent any unauthorized third parties from accessing such data.

8.4 Confidentiality agreement

All information materials and unpublished documents provided to the researcher by the sponsor shall not be disclosed to other persons or organizations without the written consent of the sponsor.

9. Revised program

Amendments to the study protocol must be discussed and agreed by the scientific committee and signed by the principal investigator and the sponsor. Important modifications and changes must be reported to the ethics committee of the group leader unit for review and agreement to the revised version in writing.

10. Reference

1. Freddie, et al. Global cancer statistics 2018: GLOBOCAN estimates of incidence and mortality worldwide for 36 cancers in 185 countries. (2018).

2. Zhang, S., Sun, K., Zheng, R., Zeng, H. & He, J.J.J.o.t.N.C.C. Cancer incidence and mortality in China, 2015. (2020).

3. Preoperative chemotherapy for non-small-cell lung cancer: a systematic review and meta-analysis of individual participant data. Lancet (London, England) 383, 1561-1571 (2014).

4. Liang, Y. & Wakelee, H.A. Adjuvant chemotherapy of completely resected early stage non-small cell lung cancer (NSCLC). Transl. Lung Cancer Res. 2, 403-410 (2013).

5. Hellmann, M.D., et al. Pathological response after neoadjuvant chemotherapy in resectable non-small-cell lung cancers: proposal for the use of major pathological response as a surrogate endpoint. Lancet Oncol. 15, e42-50 (2014).

6. Syed, Y.Y. Anlotinib: First Global Approval. Drugs 78, 1057-1062 (2018).

7. Han, B., Li, K., Zhao, Y., Wang, Q. & Wang, H.J.J.o.T.O. P2.03a-001 A Randomized Phase III Clinical Trial of Anlotinib Hydrochloride in Patients with Advanced Non-Small Cell Lung Cancer (NSCLC):Topic: Clinical Trials. 12, S886-S887 (2017).

8. Fukumura, D., Kloepper, J., Amoozgar, Z., Duda, D.G. & Jain, R.K. Enhancing cancer immunotherapy using antiangiogenics: opportunities and challenges. Nat. Rev. Clin. Oncol. 15, 325-340 (2018).

9. Ning, et al. Phase 2 trial of neoadjuvant bevacizumab plus pemetrexed and carboplatin in patients with unresectable stage III lung adenocarcinoma (GASTO 1001). 122, 740-747 (2016).

10. Yang, X., Yin, R. & Xu, L.J.N.E.J.o.M. Neoadjuvant PD-1 Blockade in Resectable Lung Cancer. 379, e14 (2018).

11. Provencio, M., et al. Neoadjuvant chemotherapy and nivolumab in resectable non-small-cell lung cancer (NADIM): an open-label, multicentre, single-arm, phase 2 trial. The Lancet Oncology 21, 1413-1422 (2020).

12. Li, B., Cui, Y., Diehn, M. & Li, R. Development and Validation of an Individualized Immune Prognostic Signature in Early-Stage Nonsquamous Non–Small Cell Lung Cancer. JAMA Oncol. 3(2017).

13. Zhang, S., Xu, P., Yuan, C. & Ou, W.J.C.J.o.L.C. Safety of Neoadjuvant Bevacizumab plus Pemetrexed and Carboplatin in Patients with IIIa Lung Adenocarcinoma. 18, 365-368 (2015).
